# Synergistic Effect of Nitrogen Doping and Textural Design on Metal-Free Carbide-Derived Carbon Electrocatalysts for the ORR

**DOI:** 10.1021/acsami.5c10307

**Published:** 2025-09-09

**Authors:** Berta Pérez-Román, F. Javier Recio, Jesús López-Sánchez, Laura Pascual, M. Alejandra Mazo, Fernando Rubio-Marcos

**Affiliations:** † 119906 Instituto de Cerámica y Vidrio (ICV-CSIC), C/Kelsen 5, 28049 Madrid, Spain; ‡ Escuela de Doctorado UAM, C/Francisco Tomás y Valiente 2, 28049 Madrid, Spain; § Departamento de Química Física Aplicada, Facultad de Ciencias, Universidad Autónoma de Madrid, C/Francisco Tomás y Valiente, 7, Cantoblanco, 28049 Madrid, Spain; ∥ Instituto de Catálisis y Petroleoquímica, 16379 CSIC, C/Marie Curie 2, 28049 Madrid, Spain

**Keywords:** nitrogen doping, carbon-based materials, carbide-derived carbon, textural properties, hydrogen peroxide, oxygen reduction reaction

## Abstract

The oxygen reduction reaction (ORR) is critical to energy conversion technologies and requires efficient catalysts for superior performance. Herein, nitrogen-doped carbide-derived carbon (N-CDC) catalysts are prepared using novel engineered molecular architectures based on polymer-derived ceramic technology. The obtained catalyst materials show a surface N concentration of >5 wt % and a hierarchically porous structure, resulting in a specific surface area of over 2000 m^2^ g^–1^. Subsequently, the electrocatalytic activity toward the ORR is studied in different media (acid, neutral, and alkaline conditions) using a rotating ring-disk electrode. The N-CDC catalysts demonstrate clear improvements in performance due to nitrogen doping in neutral and acidic media, while textural properties are crucial for the ORR activity in alkaline media. Specifically, a superior onset potential (0.8 V vs RHE) and enhanced kinetics (58 mV dec^–1^) are achieved in 0.1 M KOH. This work opens new avenues in the field of electrocatalysis, highlighting the potential of N-CDC materials and their significant advantage for the controlled synthesis of hierarchical porous and doped materials.

## Graphical abstract

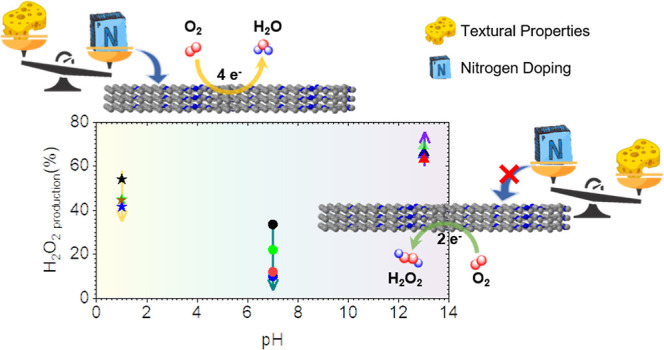

## 1 Introduction

In the current scenario, where rising energy consumption coupled with the continued use of fossil fuels is driving severe environmental challenges, the development of alternative and clean energy conversion technologies has become a top priority.^1,2^ Electrochemical energy devices, such as fuel cells and metal–air batteries, have gained significant attention for their ability to produce electrical energy by converting chemical energy through redox reactions.^3^ The oxygen reduction reaction (ORR) is an important electrochemical process occurring at the cathode of these devices, thus requiring highly effective and stable electrocatalysts to maximize the ORR efficiency.^2^

Noble metals, specifically Pt-based materials are currently highly efficient and stable catalysts for the fabrication of cathode electrodes.^4,5^ However, their shortage and high costs hinder large-scale commercialization, implying the necessity for research into alternative materials.^6^ Notably, carbon-based materials are among the most promising candidates as alternatives to high-cost Pt catalysts because of their earth abundance, acceptable cost, and high stability. In particular, porous carbon materials are well-suited for this application due to their high specific surface area (*S*_BET_) and superior electrical conductivity, providing an increase in the mass transfer and the concentration of active sites for the ORR.^7^

The electrocatalytic activity of carbon materials is strongly linked to their electronic properties, which can be effectively tailored by introducing intrinsic defects and heteroatom doping.^7^ In particular, nitrogen is one of the most studied dopants for carbon materials as its higher electronegativity and similar atomic size help promote a well-defined π-conjugation system.^8^ Interestingly, the nature and position of nitrogen within the carbon lattice induce the formation of different chemical functionalities. Reportedly, pyridinic-N, pyrrolic-N, quaternary-N (graphitic-N), and N-oxide are the fundamental nitrogen species.^9,10^ The impact of these nitrogen moieties on the ORR performance of carbon-based materials remains a topic of ongoing debate as various studies associate the influence on different nitrogen species with both 2e^–^ and 4e^–^ ORR pathways, making it difficult to stablish a clear correlation of each functionality.^11^ Additionally, distinguishing their influence from that of porous structures remains challenging. Thus, further research is needed to optimize the design of nitrogen-doped carbon materials to establish a strong correlation with oxygen reduction.

In this context, carbide-derived carbons (CDCs), defined as highly porous carbon structures obtained from metal carbide materials, stand out because their controlled synthesis route offers a tailored doping process and fine-tuning of the final porous structures.^12^ The processing of CDCs involves the selective removal of metals through etching processes, in which the adjustment of processing parameters (i.e., halogen etching atmosphere, generally chlorine, temperature, time, concentration, etc.), together with an optimum selection of the starting precursors and processing conditions, enables precise structural and microstructural control of the final CDCs.^13^ This results in the development of desired surface characteristics to improve the performance in processes like ion transport and mass transfer or the promotion of highly active sites to enhance the electrochemical reactions further. Since CDCs remain largely unexplored in the electrocatalysis field, studies have primarily focused on doping CDCs primarily with transition metals (TMs) by dual TM-nitrogen doping^13–15^ and, to a lesser extent, on metal-free doping with one or more heteroatoms (N, P, S, etc.),^16,17^ often combined with other carbon materials (such as carbon nanotubes, CNTs), yielding promising results for the ORR, especially in neutral and alkaline media.

In this study, highly porous microstructures based on metal-free nitrogen-doped CDC materials are prepared with different nitrogen functionalities. The novel dendritic structures employed possess the dual functions of providing a source of nitrogen and promoting structural control over the final CDCs. These chemical strategies are evaluated for ORR performance to gain a deeper understanding of the influence of nitrogen functionalities and textural parameters under different pH environments. The present investigation aims to expand the knowledge of N-doped CDC materials, establishing important chemical and structural correlations in the field of electrocatalysis, which remains unexplored despite their significant potential for this application.

## 2 Materials and Methods

### 2.1 Synthesis of Nitrogen-Doped CDC Catalyst Materials

Highly porous nitrogen-doped carbide-derived carbons (N-CDC) were prepared via chlorination of silicon oxycarbide (SiOC)-based precursors obtained from liquid allylhydrido polycarbosilane (AHPCS, SMP-10 Starfire Systems, USA). AHPCS was used as received and mixed with three distinct nitrogen-containing novel dendrons (D1, D2, and D3) in a 90:10 weight ratio, respectively. The dendritic structures were prepared following the synthesis procedure detailed in the Supporting Information (Section S1), in which the structural characterization was studied to confirm the intended chemical structures (Figures S1–S6), along with an analysis of their thermal behavior (Figure S7). These novel dendrons were used as nitrogen sources and to further enhance the porous microstructure of the final N-CDCs. Reactions between AHPCS and each dendritic molecule were carried out through a Schlenk line using anhydrous tetrahydrofuran (THF) as a solvent and mixing AHPCS with each dendron separately. Subsequently, 1 wt % of the platinum catalyst (platinum-1,3-divinyl-1,1,3,3-tetramethyldisiloxane (3–3.5% Pt, abcr GmbH, Germany)) was added to the reaction, and mixtures were stirred for 48 h under an Ar atmosphere to promote the cross-linking of AHPCS with the respective dendrons. After polymerization reactions, materials were pyrolyzed in an alumina tubular furnace, following a two-step thermal cycle with a heating rate of 5 °C min^–1^. Samples were heated up until 280 °C for 5 h, followed by an increase until 700 °C for 2 h to promote cross-linking and the polymer-to-ceramic transformation, respectively, resulting in N-doped SiOC structures. Subsequently, the materials were milled and sieved to less than 45 μm. The obtained powders were subjected to a chlorination treatment by placing the samples in a quartz tubular furnace and heating them to 700 °C for 2 h, with a continuous flow of Cl_2_/N_2_ of 25 mL/50 mL. Heating and cooling were performed under a N_2_ flow (100 mL min^–1^) with a heating rate of 5 °C min^–1^. Finally, the materials were subjected to a final thermal treatment at 500 °C for 4 h under a N_2_/H_2_ atmosphere to remove any chlorine trapped during the etching treatment.

The resultant N-CDC materials were labeled as 1D, 2D, and 3D, representing the carbonaceous samples obtained through the nitrogen incorporation of the different dendritic structures (D1, D2, and D3). To assess the influence of nitrogen doping, a reference sample (denoted as “Ref”) was prepared without nitrogen dendron incorporation by following the synthesis procedure described previously, except for the initial polymerization step. Instead, the AHPCS as-received was directly subjected to the pyrolysis treatment, followed by chlorination, and finished with the thermal treatment in a N_2_/H_2_ atmosphere described previously.

### 2.2 Characterization Techniques

Microstructural investigations of the prepared materials were conducted first through field emission scanning electron microscopy (FE-SEM) using Hitachi S-4700 equipment (Japan), while the elemental mapping study was carried out by energy-dispersive spectroscopy (EDS) analysis. Next, transmission electron microscopy (TEM) techniques, including high-resolution TEM (HR-TEM) and scanning TEM (STEM) in the high-angle annular dark-field (HAADF) mode, were employed to investigate the microstructural features. Imaging was performed using a JEOL JEM-2100F transmission electron microscope equipped with a field emission gun operating at 200 kV. Additionally, EDS was carried out using an Oxford INCA Energy 2000 system spectrometer attached to the same microscope.

X-ray photoelectron spectroscopy (XPS) was employed for chemical surface analysis, elucidating fundamentally the different nitrogen configurations introduced in the prepared materials. Experiments were conducted using an instrument equipped with an ultrahigh vacuum system (SPECS GmbH, Germany) and an energy analyzer (PHOI-BOS 150 9MCD). A nonmonochromatic Mg energy source (200 W—12 kV) was employed, with a sampling area of 500 × 500 mm^2^. The resulting data were calibrated by adjusting the positions to 284.6 eV (C 1s), and Shirley’s background was used as a baseline correction. Raman spectra were acquired using a confocal Raman microscope, WITec (Germany) ALPHA 300RA (Nd:YAG laser light source of 532 nm in p-polarization), recording several Raman maps in the range of 65–3850 cm^– 1^. An average spectrum of a characteristic region was presented for each sample. A 600 g mm^–1^ grating was used during measurements, as well as a laser output power of 0.7 mW to avoid overheating effects.^18^ The spectral resolution was 0.2 cm^–1^. Porosity microstructures were evaluated by obtaining the Brunauer–Emmett–Teller surface area (*S*_BET_)^19^ through a Tristar, Micromeritics analyzer (USA), using N_2_ adsorption–desorption at 77 K. Pore size analysis was estimated by the Barrett–Joyner–Halenda (BJH)^20^ method, and pore volumes were assessed in the micro-, meso- and macropore range using the single point adsorption pore volume (*V*_sp_), *V*_BJH_ desorption cumulative pore volume, and pore volume parameter up to a relative pressure of 0.99 *p*/*p*_0_. Prior to analysis, samples were degassed at 120 °C for 18 h.

### 2.3 Electrochemical Measurements

Electrochemical tests were performed on an Autolab electrochemical workstation (PGSTAT302N potentiostat, Metrohm, Switzerland) operating in a three-electrode cell. A reference electrode of Ag|AgCl (saturated KCl) regularly calibrated against a reversible hydrogen electrode (RHE) and a graphite rod were used as the counter electrode. A rotating ring disk electrode (RRDE, Pine Research Instrumentation, USA) consisting of a platinum ring and a glassy carbon disk of 0.196 cm^2^ was used as the working electrode. Catalyst inks were prepared by dispersing 5 mg of the prepared materials in 5 mL of ethanol in an ultrasonic bath for 15 min, followed by the addition of 10 μL of Nafion solution (DUPONT DE520, Ion Power, Inc., USA). Finally, the solutions were sonicated for 30 s. Prior to modification of the glassy carbon, it was cleaned by polishing with alumina powder (0.05 μm) and washed with Milli-Q water. Then, 10 μL of the solution was deposited into the glassy carbon by drop casting, followed by a drying treatment with a gentle N_2_ flow. The electrochemical performance of the studied materials was evaluated in alkaline media (0.1 M KOH), neutral phosphate buffer solution (PB) (0.1 M NaH_2_PO_4_/Na_2_HPO_4_), and acidic media (0.1 M H_2_SO_4_). All the potential values are reported to the RHE, using the expression *E*_RHE_ = *E*_Ag/AgCl_ + 0.0592 pH + 0.199 V. All of the electrochemical measurements were recorded without *iR* compensation, so data are presented as measured. The characterization of carbon materials was performed by cyclic voltammetry (CV) in N_2_-saturated media in the potential ranges of −0.15 to 0.25 V, −0.10 to 0.30 V, and −0.25 to 0.15 V (vs RHE) in the acid, neutral, and alkaline media, respectively. The electrochemically active surface area (ECSA) was determined by estimating the electrochemical double-layer capacitances (*C*_dl_) from the CV curves following eq 1. For this calculation, varied CV measurements were acquired at 5, 10, 20, 50, and 100 mV s^–1^ in the non-Faradaic potential ranges described previously. In eq 1, *i*_a_ and *i*_c_ represent the anodic and cathodic currents, respectively, while *v* is the scan rate employed for the CV measurements. Representing the linear plot of the capacitive currents vs the scan rate, *C*_dl_ can be estimated and finally converted into the ECSA following eq 2. *C*_s_ represents the specific capacitance, and the typical value for an atomically smooth planar surfaces of 0.04 mF cm^–2^ was used.^21^

1 $$\frac{\Delta i}{2}=\frac{i_{a}-i_{c}}{2}=C_{dl}\times v$$

2 $$ECSA=\frac{C_{dl}}{C_{s}}$$

The electrocatalytic activity for the ORR was studied by linear sweep voltammetry (LSV) in the different O_2_-saturated media at 5 mV s^–1^ and using a rotating speed of 1600 rpm. A constant potential of 1.2, 1.6, and 1.4 V vs RHE was applied to the Pt ring electrode in alkaline, neutral, and acid media, respectively.

Hydrogen peroxide (H_2_O_2_) selectivity was calculated based on the disk (*I*_D_) and ring currents (*I*_R_) of the working electrode, as presented in eq 3, while the exact number of transferred electrons (*n*) was assessed according to the Koutecky–Levich equation (eq 4), where *N* represents the ring collection efficiency, set at 0.38.

$$Selectivity\;\left(\%\right)=200\frac{\frac{I_{R}}{N}}{\frac{I_{R}}{N}+I_{D}}$$ 3

4 $$n=4\frac{I_{D}}{\left(I_{D+}\frac{I_{R}}{N}\right)}$$

The stability of the catalysts was evaluated by an accelerated degradation test, comparing LSV curves recorded at a rotation rate of 1600 rpm before and after 1000 CV cycles conducted at a scan rate of 100 mV s^–1^ in O_2_-saturated electrolytes. The CV measurements were performed within potential windows of 0.5–0.9, 0.4–0.8, and 0.2–0.6 V vs RHE for 0.1 M KOH, 0.1 M PB, and 0.1 M H_2_SO_4_ electrolytes, respectively.

## 3 Results and Discussion

### 3.1 Characterization of the Prepared CDC Materials

Figure 1a illustrates the main steps of N-CDC processing: first, the polymerization reaction between a commercial AHPCS and the designed dendrons (D1, D2, and D3); second, the pyrolysis treatment to obtain a polymer-derived ceramic structure; and finally, chlorination at elevated temperature to promote the hierarchically porous structures (1D, 2D, and 3D), where the nitrogen doping is successfully incorporated by each dendritic molecule. The microstructural properties of the samples are investigated by SEM and TEM microscopic studies. In the case of the reference material (Ref), uneven shaped particles are predominantly observed. The SEM image of Figure 1b shows a representative example where the particle is essentially composed of carbon, as evidenced by the corresponding elemental map (Figure 1c). In detail, the particle is formed by a set of laminar structures, as seen in Figure 1d, due to the chlorination process used to prepare the final CDCs and, more specifically, due to the remaining carbonaceous composition without any visible surface defects.

**Figure 1:**
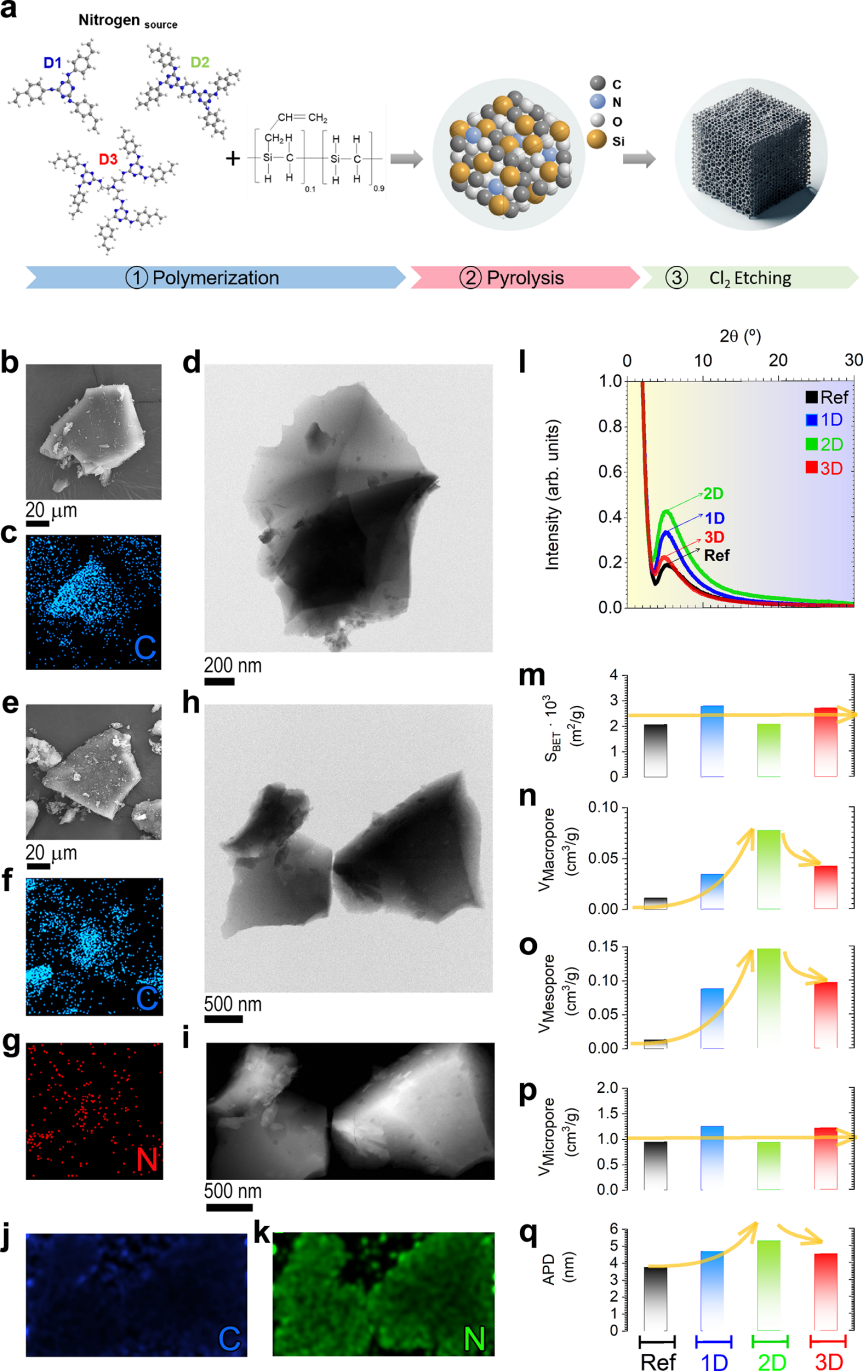
Microstructural investigations and textural study. (a) Graphical representation of the preparation route for the synthesis of N-CDC materials. **Ref sample**: (b) SEM image, (c) elemental mapping of C, and (d) TEM micrograph of the Ref sample. **2D material**: (e) SEM image and elemental mapping of (f) C and (g) N corresponding to figure (e). (h) TEM and (i) HAADF images and elemental mapping of (j) C and (k) N corresponding to figure (i). **Comparison between Ref, 1D, 2D, and 3D samples**: (l) XRD patterns. (m) *S*_BET_, (n) *V*_macro_, (o) *V*_meso_, (p) *V*_micro_, and (q) average pore diameter (APD) obtained by N_2_ adsorption–desorption measurements (4 V/A).

Moving on to the nitrogen-doped materials, Figure 1e shows the SEM micrograph of the 2D material, in which similarly shaped particles are found as in the Ref material. The presence of carbon and nitrogen (Figure 1f,g) is detected in the 2D sample, evidencing the homogeneous incorporation of nitrogen through the designed dendritic structure (D2) used as the nitrogen source. The sample is examined by TEM/STEM microscopy, where the low-magnification TEM and STEM-HAAFD micrographs of the 2D material are shown in Figure 1h,i, with the corresponding carbon and nitrogen elemental maps (Figure 1j,k), respectively, to further confirm the introduction of nitrogen through the synthesis procedure described earlier. Additional microstructural information on the 2D sample, coupled with the examination of the 1D and 3D materials by SEM and TEM microscopies, is displayed in Figure S8a–s. Nitrogen is detected uniformly in the different nitrogen-doped samples, as shown in the elemental mapping presented in Figure S8, thereby confirming a successful and uniform nitrogen distribution obtained by doping the materials with the different prepared dendrons. A detailed examination of the surface morphology was carried out by SEM microscopy, which revealed an increased roughness surface in the 2D sample (Figure S8m), compared to that in the Ref, 1D, and 3D materials (Figure S8c,h,r, respectively). This rough surface can be attributed to some extent to the chlorination process used to etch the structures. However, the notable presence in the 2D sample compared to the rest of the CDC materials suggests an increased defect incorporation (i.e., porous formation) with the D2 dendritic structure compared to the other dendrons. This phenomenon will be further evaluated using the N_2_ adsorption–desorption technique and Raman and XPS spectroscopies.

The phase study of the prepared materials is carried out by XRD analysis, with the corresponding diffraction patterns shown in Figure 1l. As expected, the materials are highly amorphous as noncrystalline peaks related to graphite or other carbon-based phases are detected in the spectra, consistent with the low-temperature treatment used during the preparation route. In the low 2θ ° region, a broad shoulder located at around 2θ = 5° is detected in all the samples, showing an increased relative intensity in the N-CDCs and reaching its maximum in the 2D sample.

This peak indicates the presence of a larger porous microstructure, which is particularly pronounced in the N-CDCs, suggesting enhanced porosity formation due to the introduction of the dendritic structures, particularly evident for the D2 dendron. The result is further verified by textural analysis. Figure S9 displays the N_2_ adsorption–desorption curves, showing type-Ia for the Ref sample and type-IVa for the N-doped materials.^22^ The former is characterized by the increased adsorbed volume in the very first region of relative pressures, which is characteristic of microporous materials. On the other hand, the N-doped samples display hysteresis cycles, particularly pronounced in the 2D material, indicating the formation of mesopores, together with a moderate increase in N_2_ adsorption at higher relative pressures, suggesting the additional presence of macropores. The larger pores observed in the 2D sample can be tentatively attributed to partial dendron fragmentation occurring during the initial cross-linking phase at 280 °C, as supported by the TG–DTA analysis (Figure S7). This early structural disruption promotes the formation of internal voids, which subsequently evolve into larger pores during pyrolysis and chlorination.

Thus, the dendritic structures induce the development of hierarchical porous microstructures, with the 2D sample showing a significant increase in meso- and macroporosity, in agreement with the X-ray diffraction patterns shown in Figure 1l. These porous structures result in *S*_BET_ values of 2057 and 2073 m^2^ g^–1^ for the Ref and 2D samples, respectively, and superior *S*_BET_ values of 2783 and 2696 m^2^ g^–1^ for the 1D and 3D samples, respectively (Figure 1m).

To gain a deeper insight into the porous microstructure, the macro-, meso-, and micropore volumes are estimated and compared across the prepared materials, as depicted in Figure 1n–p. The average pore diameter (APD) is also plotted in Figure 1q. As demonstrated in Figure 1n,o, elevated *V*_meso_ and *V*_macro_ are observed in the N-doped samples, with the 2D material exhibiting particularly pronounced effects, reaching values of *V*_meso_ = 0.15 g cm^–3^ and *V*_macro_ = 0.08 g cm^–3^. Conversely, lower values are recorded in both 1D and 3D materials (see Figure 1n,o). The microporosity is predominantly attributable to the etching of carbide-based structures derived from the polymeric precursor, as evidenced by the elevated *V*_micro_ observed in the Ref material (Figure 1p). Furthermore, the formation process is significantly enhanced by the presence of D1 and D3 molecules, as noted by the superior *V*_micro_ values obtained in both materials, which are consistent with the superior *S*_BET_ values observed in these samples. Therefore, greater APD values are obtained in the 2D sample (5.3 nm), followed by 1D (4.7 nm), 3D (4.5 nm), and Ref material (3.8 nm) (Figure 1q), respectively.

The surface compositional analysis is performed by XPS to corroborate the successful chlorination treatment and the effective nitrogen doping promoted by the dendritic molecules. As illustrated in Figure 2a–d, Table S1 comprises the surface compositional analysis obtained from the survey spectra. The analysis reveals the presence of photoelectron peaks and Auger bands associated with C, N, O, Si, and residual traces of Cl. As anticipated, the composition of surface materials is predominantly carbon, exhibiting concentrations exceeding 87 wt %, accompanied by minimal silicon content (<1 wt %) for the Ref, 1D, and 3D materials. This phenomenon is indicative of the efficacy of the chlorination treatment, which has successfully removed the carbide phase, as previously hypothesized by the elevated pore microstructures displayed in Figure S9. Furthermore, comparable oxygen concentrations (approximately 6 wt %) are detected in Ref, 1D, and 3D materials, attributed to a slight surface oxidation of the structures through the thermal treatments.^23^ In contrast, the 2D material exhibits an augmented presence of Si and O, accompanied by a diminished carbon concentration, suggesting less effective etching and a heightened presence of the residual SiOC phase (comprising disparate SiO_*x*_C_4–*x*_ units, where *x* varies from 4 to 0). Similar Cl traces remaining in the samples (i.e., traces amounts less than 1 wt %) are also detected for the varied samples. Regarding nitrogen content, comparable concentrations ranging from 5.2 to 5.6 wt % are found in the N-CDC materials, thereby confirming the successful introduction of nitrogen heteroatoms through the three dendrons, as previously observed by SEM and TEM investigations (see Figures 1 and S8).

**Figure 2:**
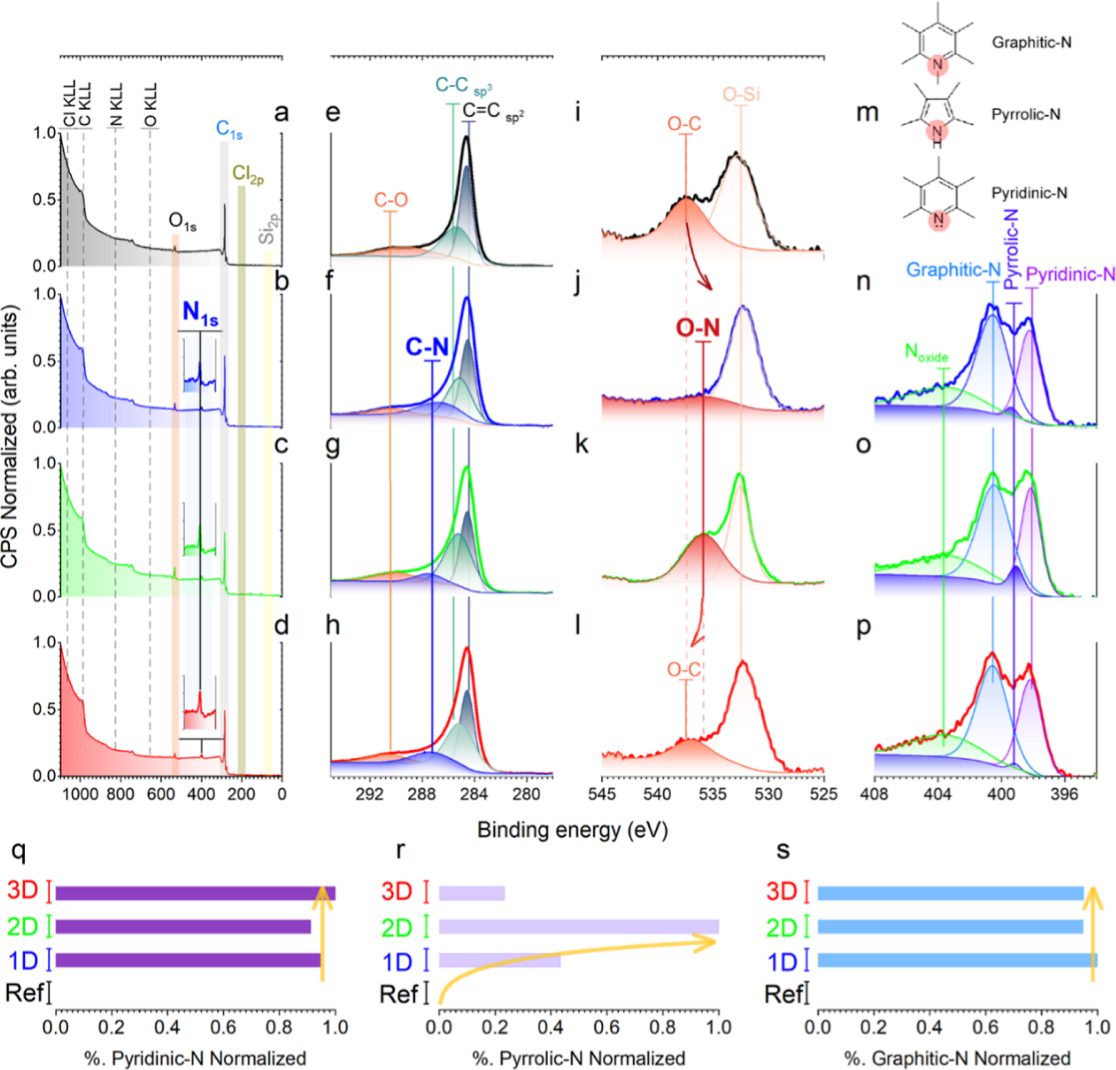
Revealing surface chemical bonding states by XPS. Survey spectra including the electronic core levels (Si 2p, C 1s, N 1s, Cl 2p, and O 1s) and the Auger bands, C 1s and O 1s spectra, and their corresponding Gaussian deconvolution of (a,e,i) Ref, (b,f,j) 1D, (c,g,k) 2D, and (d,h,l) 3D CDC materials, respectively. (m) Representative schemes of the graphitic-N, pyrrolic-N, and pyridinic-N functionalities. N 1s spectra of the N-CDC materials: (n) 1D, (o) 2D, and (p) 3D. Normalized relative concentrations of the characteristic nitrogen functionalities of the N 1s spectra: (q) pyridinic-N, (r) pyrrolic-N, and (s) graphitic-N.

To unveil the C-bonding environments, C 1s core level high-resolution spectra (Figure 2e–h) are deconvoluted into four different peaks located at approximately 284.6 (C sp^2^), 285.1 (C sp^3^), 287.2 (C–N), and 290.1 eV (C–O).^24^ The Ref sample is fitted by equivalent bands, with the exception of the C–N-related peak (Figure 2e), with this band providing evidence regarding the nitrogen incorporation into the N-doped materials. Despite the challenge of distinguishing between the C sp^2^ and C sp^3^ bands due to the similarities in their binding energies, a growing presence of C sp^3^ is observed in the N-CDC samples compared to the Ref material, as illustrated in Figure 2e–h. This increase is attributed to the introduction of the dendritic structures, suggesting an induced defect incorporation toward the designed molecular architectures.^25^ Consequently, a tentative correlation between both carbon hybridization states is calculated (Table S2).

Generally speaking, a higher C sp^3^/C sp^2^ ratio suggests an increased presence of topological defects in the carbon structures, such as vacancies, incorporated heteroatoms, and/or pentagonal defect carbons.^24,26^ Therefore, the enhanced C sp^3^/C sp^2^ ratio observed in the N-doped samples proves an augmented incorporation of defects during the nitrogen doping process, a phenomenon that is particularly pronounced in the 2D sample. This observation is in alignment with the earlier findings from SEM and TEM investigations (Figures 1 and S8m). In addition, the O 1s spectra and their respective deconvolutions are presented in Figure 2i–l, discerning the presence of two distinct peaks located at approximately 532.5 and 536.5 eV, respectively. The former is associated with O–Si,^23,27^ and the latter can be attributed to either O=C or O–N functionalities.^28^

A more profound investigation into the nitrogen configurations (Figure 2m) is also conducted by deconvoluting the high-resolution N 1s spectra, as presented in Figure 2n–p. The spectra are fitted by four Gaussian bands, centered at 398.2, 399.2, 400.5, and 403.6 eV attributed to pyridinic-N, pyrrolic-N, graphitic-N, and N-oxide bonds, respectively.^29,30^ The relative concentrations of the various nitrogen species are displayed in Table 1, which demonstrates that nitrogen is fundamentally introduced as graphitic-N (45–48%), followed by pyridinic-N (29–32%), N-oxide (20–22%), and pyrrolic-N (1–5%). As illustrated in Figure 2q–s, samples 1D and 3D exhibit comparable spectra and relative concentrations, including the pyrrolic-N content. However, a higher concentration is observed in the 1D material, reaching ∼2%. This effect is more pronounced in the 2D sample, reaching the highest concentration of pyrrolic-N (4.5%) (Table 1), which could be tentatively derived from the N-amines presented in the starting D2 dendron as –NH– bonds and −N– of the piperazine ring. These features could promote cyclization reactions to form the pyrrolic-N bond.^31^ In addition, the increased presence of pyrrolic-N can be linked to the major formation of defects detected in the 2D material as nitrogen is in a 5-membered ring, thereby promoting major disruption of the carbon structures compared to the graphitic-N and pyridinic-N functionalities.^25^

**Table 1:** Textural Properties and Nitrogen Surface Composition of the Prepared Materials^a^

	Textural properties					Surface concentration obtained by XPS				Defect concentration
Catalysts	*S*_BET_ (m^2^ g^–1^)	*V*_micro_	*V*_meso_	*V*_macro_	N (wt %)	Pyridinic-N (%)	Graphitic-N (%)	N-oxide (%)	Pyrrolic-N (%)	*I*_D_/*I*_G_
		(cm^3^ g^–1^)								
Ref	2057	0.95	0.02	0.01						1.44
1D	2783	1.25	0.09	0.03	5.2	30.2	47.8	20.0	2.0	1.33
2D	2073	0.93	0.15	0.08	5.6	29.1	45.3	21.1	4.5	1.51
3D	2696	1.21	0.10	0.04	5.5	31.9	45.4	21.7	1.0	1.22

a Summary of the most important microstructural parameters of the prepared materials, including detailed information about the porous microstructures, nitrogen concentration and functionalities studied by XPS, and defect concentration evaluated by Raman spectroscopy.

The investigation of carbon-based materials is conducted through confocal Raman microscopy, a technique that facilitates the elucidation of the degree of order and the structural defects generated during the etching treatment. The analysis is further augmented by the introduction of dendritic molecules, which serve to enhance the complexity of the materials under study. Figure S10a–d displays the average spectra obtained from the Raman maps acquired from the different CDC samples, showing a highly disordered carbon phase due to the low pyrolysis temperature employed during the polymer-to-ceramic transformation, which is in accordance with previous studies.^32–34^ In order to extract structural correlations, a Voigt fitting is performed with four contributions; the bands D and G centered at around 1340 and 1600 cm^–1^ ^35^ and two broad contributions located at 1220 and 1530 cm^–1^, named in the literature as band D* and D″, respectively. The D band is activated by the presence of structural defects, and the G band corresponds to the in-plane vibration of sp^2^-hybridized carbon atoms in graphitic structures. In turn, the D* band is attributed to aliphatic or amorphous structures, i.e., carbon atoms outside the perfect planar carbon network.^36^ Conversely, band D″ is associated with the distortion of carbon-based structures, which may result from the presence of heteroatoms or topological defects as carbon pentagon structures. Nevertheless, this attribution remains a topic of ongoing discussion.^26,30^

The intensity ratio of bands D and G (*I*_D_/*I*_G_) of the prepared materials is calculated (see Table S3) and illustrated in Figure S10e and Table 1. The maximum values are observed for the Ref and 2D materials, indicating a significant presence of defects in the sp^2^ network of carbon structures. In contrast, 1D and 3D samples show reduced *I*_D_/*I*_G_ values (see Table S3), which may be ascribed to the capacity of the dendrons to form a specific structural arrangement within the sp^2^ bonds of the resulting N-CDC materials. The observed effect is attributable to the engineered architecture of the molecules employed for nitrogen incorporation.

To conclude with the characterization section, Table 1 summarizes the most significant textural parameters and the nitrogen surface concentration.

This includes the various nitrogen functionalities, in conjunction with the characteristics of the hierarchically porous structures developed, demonstrating the effectiveness of our synthetic strategy, which allows homogeneous doping and avoids the pore-blocking effects that may arise from postsynthetic doping methods. In this context, all materials show an elevated *S*_BET_ (>2000 m^2^ g^–1^), which may influence the ORR performance. In addition, the presence of diverse pore volumes, ranging from micro- and meso- to macropores, can add a certain degree of textural complexity. In this sense, micropores ensure a greater number of active sites, while mesopores reduce the mass-transport losses and enable access of the oxygen molecule to the active sites.^16,37^ Consequently, the development of a hierarchical porous microstructure catalyst is imperative to ensuring optimal ORR performance. Furthermore, the varying nitrogen functionalities introduced will perform distinct roles in the context of the ORR, which is highly dependent on the pH of the medium. The subsequent section addresses the impact of the textural properties in conjunction with nitrogen doping on electrocatalytic activity.

### 3.2 Electrochemical Properties Evaluation

An exhaustive electrochemical characterization of the N-CDC materials is conducted in various pH media to evaluate the influence of the microstructural parameters. CV measurements are carried out to estimate the *C*_dl_ (eq 1) and ECSA (eq 2) parameters and their dependence on the different pH environments. Figure 3a displays the voltammograms performed in neutral media for the 2D catalyst, while the CV plots of the rest of materials (including the Ref and N-doped CDC samples) and their response in neutral, alkaline, and acid media are displayed in Figures S11–S13. It is evident from the results that all of the prepared materials exhibit non-Faradaic behavior in the potential studied range, as well as at different scan rates. The *C*_dl_ and ECSA parameters are subsequently estimated for the various electrocatalysts at a constant potential of 0.05 V vs RHE. Figure 3b–d illustrates the linear plots calculated for neutral, acid, and alkaline conditions, respectively. The electrochemical parameters obtained are summarized in Table 2.

**Figure 3:**
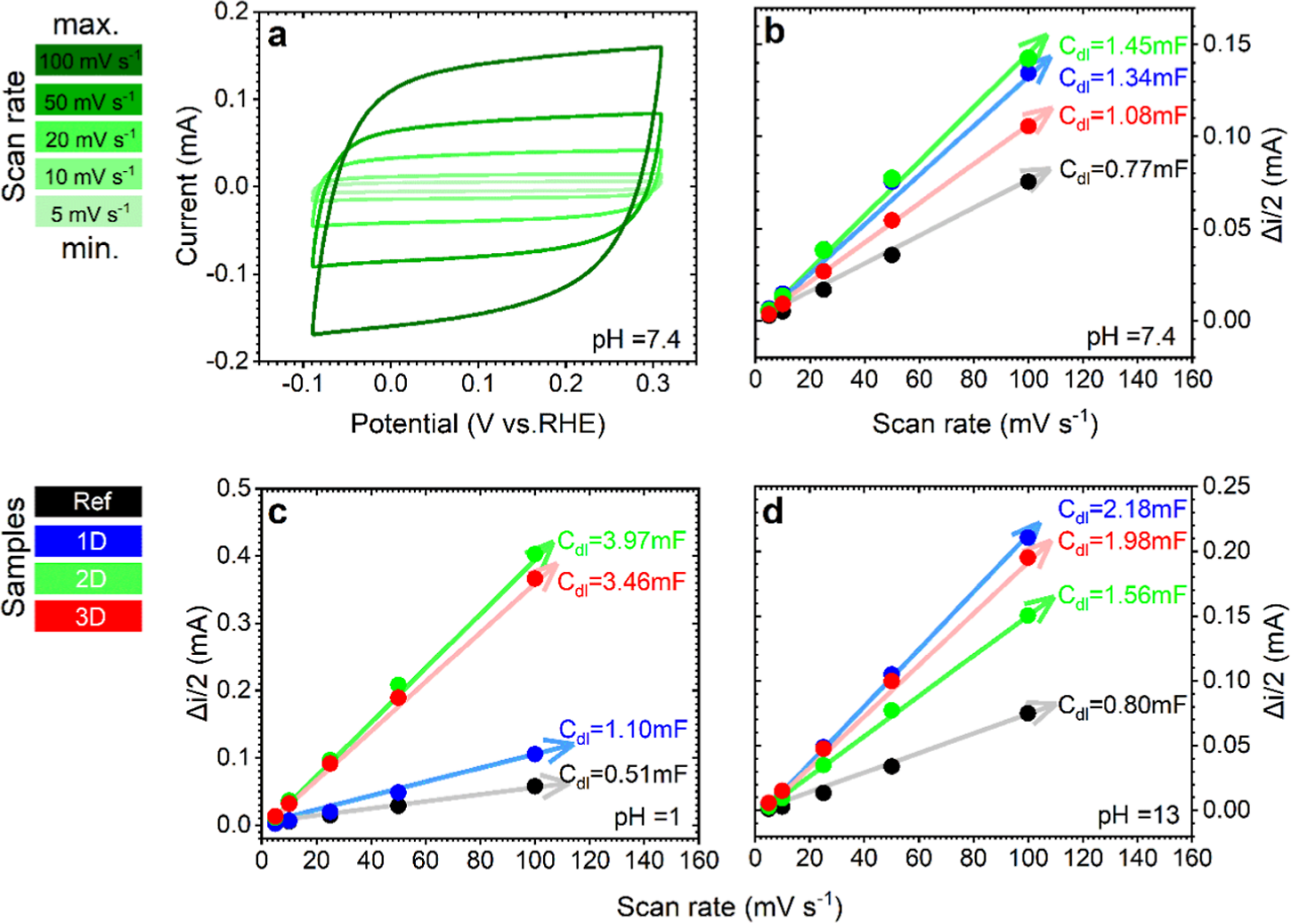
Electrochemical characterization by CV measurements and ECSA calculation. (a) CV curves of the 2D catalyst recorded at scan rates of 5, 10, 20, 50, and 100 mV s^–1^ in N_2_-saturated 0.1 M PB. Linear plotting of the average capacitive current (Δ*i*) vs scan rates to obtain the *C*_dl_ values of the different materials in (b) 0.1 M PB, (c) 0.1 M H_2_SO_4_, and (d) 0.1 M KOH media. Notice that CV curves were recorded in the potential window of approximately −0.1 to +0.3 V vs RHE.

**Table 2:** Electrochemical Parameters Obtained from the CV and LSV Measurements in Different Media^a^

Catalysts	*E*_ONSET_ (V vs RHE)	*E*_1/2_ (V vs RHE)	*n*	H_2_O_2_ (%)	*C*_dl_ (mF)	ECSA (cm^2^)	*E*_ONSET_^*^ (V vs RHE)
**0.1 M KOH**							
Ref	0.77	0.69	2.6	66	0.80	20.1	0.69
1D	0.80	0.68	2.6	65	2.18	54.5	0.75
2D	0.80	0.68	2.6	69	1.56	39.0	0.78
3D	0.76	0.62	2.7	63	1.98	49.5	0.74
**0.1 M PB**							
Ref	0.48	0.03	3.3	34	0.77	19.2	0.38
1D	0.64	0.31	3.8	10	1.34	33.5	0.54
2D	0.61	0.36	3.6	22	1.45	36.3	0.54
3D	0.65	0.35	3.8	12	1.08	27.0	0.57
**0.1 M H_2_SO_4_**							
Ref	0.28	0.22	2.9	54	0.51	12.8	0.08
1D	0.40	0.26	3.2	42	1.10	27.5	0.22
2D	0.40	0.26	3.1	45	3.97	99.3	0.24
3D	0.44	0.25	3.1	44	3.46	86.5	0.27

a The table summarizes the most important electrochemical parameters including *E*_ONSET_, *E*_1/2_, *n*, H_2_O_2_ selectivity, *C*_dl_, and ECSA parameters, together with *E*_ONSET_^*^_,_ representing the values after degradation measurements. The H_2_O_2_ production is collected at 0.45, −0.20, and 0.00 V vs RHE for alkaline, neutral, and acidic media, respectively.

Across a range of pH values, the N-CDC materials possess noticeably elevated ECSA values in comparison to the Ref sample (Figure 3b–d), thereby highlighting the beneficial effect of nitrogen doping and the enhanced porosity on the active surface area. With regard to the reference material, ECSA increases with the pH, reaching a maximum of 20.1 cm^2^ in 0.1 M KOH (see Table 2). A comparable trend is observed for the 1D sample, which attained a maximum ECSA of 54.5 cm^2^ in the alkaline media. Conversely, the 2D and 3D materials exhibit their maximum ECSAs in acidic media, attaining approximately 99.3 and 86.5 cm^2^, respectively. As the nitrogen content and the relative concentrations of the nitrogen functionalities exhibit no significant differences between the N-CDC samples (Table 1), variations in ECSA across the samples may be attributed to their textural properties. In an alkaline environment (0.1 M KOH), OH^–^ ions are the predominant agents in the formation of the electrical double layer. This predominance arises from their primary interaction with the microporosity structure that characterizes such environments. In contrast, in the PB buffer and H_2_SO_4_ media, ions such as SO_4_^2–^ and H_2_PO_4_^–^ together with HPO_4_^2–^ ions, respectively, play a key role in the double layer formation. The latter ions possess larger ionic radii in comparison to OH^–^, thus resulting in more effective interaction with meso- and macroporosity as opposed to microporosity.^38–40^ Therefore, according to Figure 1n,o and Table 1, the elevated *V*_meso_ and *V*_macro_ values attained in the 2D sample are responsible for the enhanced ECSAs observed in the neutral (Figure 3b) and acid media (Figure 3c). In contrast, the higher *V*_micro_ found in the 1D and 3D materials promotes an increase in the ECSA values on the alkaline media (see Figure 3d). This leads to superior *C*_dl_ and ECSA in the 1D sample (*V*_micro_ = 1.25 cm^3^ g^–1^), followed by the 3D material (*V*_micro_ = 1.21 cm^3^ g^–1^). This explanation is probably due to the enhanced interaction of the OH^–^ ions within the porous micropore range. Thus, it can be established that the ECSA parameter in these newly synthesized N-CDC materials is primarily governed by their hierarchical porosity, demonstrating an important effect of microporosity in 0.1 M KOH and meso–macroporosity in 0.1 M H_2_SO_4_.^41^

The electrocatalytic activity for the ORR is studied by LSV measurements with a RRDE equipment. Figure 4 displays the ORR polarization curves, the Tafel slopes, their corresponding H_2_O_2_ selectivity, and the electron transferred number (*n*) of the designed materials recorded in both neutral (Figure 4a–d) and alkaline media (Figure 4e–h). Figure S14 displays the information obtained from the acidic environment. Alongside, Table 2 collects the electrochemical results obtained in the different pH media. All catalysts present good electrocatalytic activity for the ORR. In the context of neutral media, the polarization curve (Figure 4a) of the Ref sample exhibits a lower level of catalytic activity in comparison to that of the N-CDC materials. As observed, the onset potential of the N-doped catalysts shifts to more positive values, from the Ref material (*E*_ONSET_ = 0.48 V vs RHE) to the N-CDCs (*E*_ONSET_ > 0.60 V vs RHE) (Table 2), indicating an improvement of the catalytic activity due to the nitrogen doping. The enhanced catalytic activity resulting from the nitrogen introduction is also observed in the half-wave potential (*E*_1/2_) and the limiting current (*J*_L_) (Figure S15a), obtaining similar values among the N-doped materials, including the onset potentials. These features are indicative of enhanced catalytic efficiency and altered selectivity, a phenomenon that can be ascribed to the stronger interaction where the nitrogen heteroatoms and the hierarchically porous structure predominate. In addition, the Tafel plots indicate that the CDC materials present a uniform mechanism for the ORR, exhibiting values that approximate 120 mV dec^–1^. This observation suggests that the initial electron transfer is the rate-determining step for all of the samples. Furthermore, there is also an increase in the selectivity of the N-CDC materials for the ORR (Figure 4c,d). The undoped carbon catalyst (Ref sample) presents a mixed mechanism via 2 and 4e^–^ with a 30% production of H_2_O_2_ during the ORR. However, the N-doped materials show an electroreduction with a H_2_O_2_ production lower than 20%, which implies a direct 4e^–^ electroreduction to H_2_O. The phenomenon is noticeable in 1D and 3D catalysts due to the limited H_2_O_2_ produced (10 and 12%, respectively) and the high number of electrons transferred (*n* ∼ 3.8) (see Table 2). Therefore, the improvement in selectivity may be associated with the synergistic effect of nitrogen doping and textural properties. The elevated H_2_O_2_ production observed for the 2D catalyst can be tentatively ascribed to its slightly higher oxygen and silicon content, as revealed by XPS (Table S1), which may interfere with the reaction mechanism or reduce active site accessibility under neutral pH conditions.

**Figure 4:**
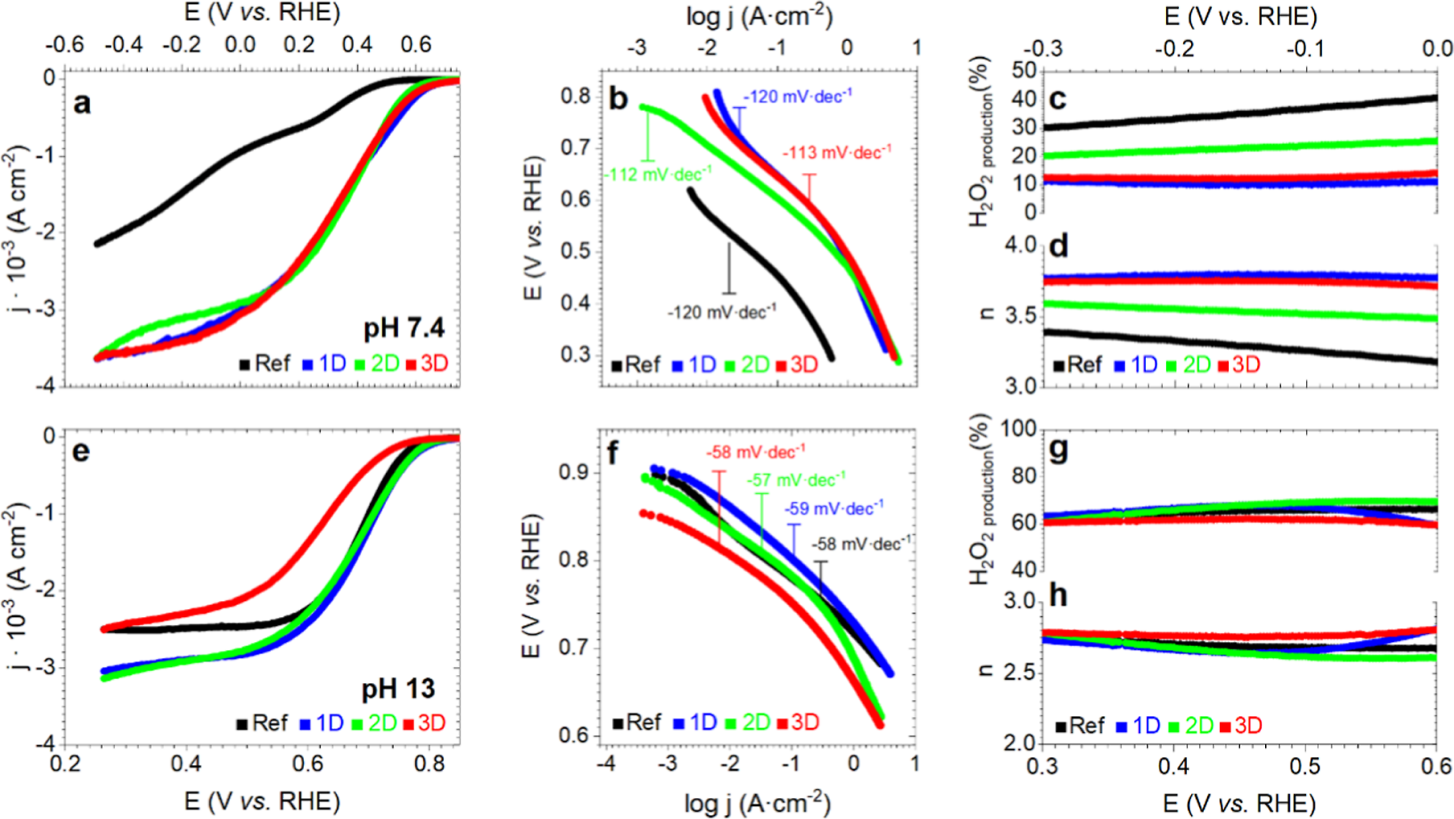
Electrocatalytic ORR performance in neutral and alkaline media. LSV, Tafel slopes, and *n*/H_2_O_2_ production calculated by Koutecky–Levich analysis of the Ref and N-CDC materials in an O_2_-saturated (a–d) neutral medium (0.1 M PB solution) and (e–h) alkaline environment (0.1 M KOH), respectively. The LSV curves are recorded in a RRDE with a rotation rate of 1600 rpm and at scan rate of 5 mV s^–1^. The constant ring potential applied is 1.6 and 1.2 V vs RHE for the neutral and alkaline media, respectively.

In consideration of the presence of various nitrogen functionalities within the obtained N-doped materials and the apparently contradictory results reported in the literature concerning the most effective nitrogen functionality, it is challenging to establish a direct correlation between the effect of a specific nitrogen moiety and the enhanced activity observed in the N-doped CDC.^11^ Nevertheless, it appears that pyridinic-N and graphitic-N exhibit the highest catalytic activity, while pyrrolic-N, although it also contributes, is the least explored N configuration in ORR experiments and exerts a comparatively minor effect on the ORR.^42–44^ Some studies stablished that pyridinic-N acts as active sites for the ORR, by helping capture HPO_4_^2–^ and H_2_PO_4_^–^protons, due to its charge neutralization capability.^44,45^ Consequently, the slightly increased pyridinic-N found in 1D and 3D samples and its coexistence with graphitic-N, together with the enhanced porous microstructure, specifically the increased *V*_micro_ detected in 1D and 3D materials, which leads in superior *S*_BET_, suggest that both parameters play an important role in the ORR performance of the prepared materials in neutral media.

Figure 4e shows the LSV curves obtained in the alkaline medium, displaying polarization curves with well-defined diffusion-limited current plateaus for all of the catalysts. The electrocatalytic activity in alkaline medium increases with respect to the neutral environment. Samples 1D and 2D exhibit an *E*_ONSET_ of approximately 0.8 V, while 3D and Ref materials show slightly lower onset potentials (Table 2). Additionally, considering the Tafel slopes (Figure 4f), a similar ORR mechanism is detected across the different samples, reaching values close to the theoretical one of 57–59 mV dec^–1^, strongly indicating an efficient ORR in the developed catalysts. In contrast to the neutral medium, no significant improvements in the selectivity of the ORR for the N-doped materials are noted, and the catalysts present a number of electrons transferred lower than 3 (*n* = 2.7). This behavior reveals a mixed mechanism with a majority of 2e^–^ pathway, in which a large amount of peroxide is electrogenerated in the form of HO_2_^–^ (p*K*_a_(H_2_O_2_) = 11.6). The comparable ORR performance obtained in 0.1 M KOH for both the Ref and the N-doped samples strongly suggests that the parameter governing the oxygen reduction activity of these CDCs in alkaline media is primarily their textural properties rather than nitrogen doping, as no remarkable variations in ORR activities are detected in the N-doped materials (Table 2 and Figure 4g,h).

The electrochemical performance of the novel materials is also evaluated in 0.1 M H_2_SO_4_ (Figure S14). The analysis reveals lower catalytic activities than in neutral and alkaline media, as evidenced by the lower onset potential values (Table 2 and Figure S14a). In accordance with the neutral medium, an effect of nitrogen doping is discerned in the onset potentials, as reflected by the improvement of the *E*_ONSET_ from the Ref material toward N-doped CDCs (Figure S14a). Tafel slopes show poor kinetic activity in this medium (Figure S14b). A mixed mechanism involving 2 and 4e^–^ is ascertained, attributed to the electrons transferred in proximity to 3, along with a H_2_O_2_ production of approximately 45% for the N-doped catalysts. The evaluation of the n and the H_2_O_2_ selectivity demonstrates that the increase in the N-doped catalysts is primarily observed at low overpotentials (Figure S14c,d), as in the neutral medium, but with a lower number of electrons transferred and higher H_2_O_2_ production. Therefore, the designed catalysts exhibit the least favorable ORR activity in the acidic media, remaining below the values reported in the literature (Table 3). This poor performance is fundamentally attributed to the slower reaction kinetics in acidic media, combined with possible degradation or blockage of the active sites of CDC structures.^46^ Alongside, the protonation of pyridinic-N sites (p*K*_a_ ≈ 6.5), which convert to the pyridinium form under acidic conditions (pH 1), hinders O_2_ adsorption, suppressing ORR activity.^9,47^

**Table 3:** Summary of ORR Performances of N-Doped Carbon-Based Materials and a Benchmark Pt/C Reported in the Literature^a^

Material	Electrolyte	N (at. %)	*S*_BET_ (m^2^ g^–1^)	Method	*E*_ONSET_ (V vs RHE)	*n*	H_2_O_2_ (%)	Tafel (mV dec^–1^)	Ref
**Alkaline Media**									
Pt/C	0.1 M KOH			RDE	1.04	4.0		–70	^59^
P-NMG-5	0.1 M KOH	8.7	243	RRDE	<0.70	2.6–2.7	∼63		^60^
N-C900	0.1 M KOH	1.8	1106	RRDE	0.67	∼3	56.7	–65	^61^
N-CW30	0.1 M KOH	3.6	1270		0.86	3.2		–68	^62^
N-CDC (13N)	0.1 M KOH	3.6	1988	RDE	0.91	∼4			^52^
N-CDC/SiC	0.1 M KOH	5.9		RDE		3.8		–56	^58^
N-CDC/CNT mel	0.1 M KOH	3.2	408	RRDE	0.91	3.6	∼20		^63^
1D	0.1 M KOH	4.6	2783	RRDE	0.80	2.6	68	–59	This work
**Neutral Media**									
Pt/C	0.01 M PBS			RDE				–137	^59^
N-HPCs	0.01 M PBS	2.4	382	RDE	0.64	∼4		–116	^59^
N-AC	0.1 M PBS	1.6	797	RRDE	0.85	3.8	<10		^44^
1D	0.1 M PB	4.6	2783	RRDE	0.64	3.8	10	–120	This work
**Acidic Media**									
Pt/C	0.5 M H_2_SO_4_			RDE	0.79	4.0		–77	^59^
NC-800	0.5 M H_2_SO_4_	10.7	658	RRDE	0.60	3.8	6.8	–314	^61^
N-GNP	0.5 M H_2_SO_4_	3.3	762	RDE	0.48	3.9			^9^
1D	0.1 M H_2_SO_4_	4.6	2783	RRDE	0.40	3.2	42	–96	This work

a This table collects information about the nitrogen content included in the materials, together with S_BET_ parameter (except for the benchmark Pt/C catalyst), and the electrochemical performance of different carbon materials (method, *E*_ONSET,_ *n*, H_2_O_2_ and Tafel slope). Notice that the RRDE and RDE mean rotating-ring disk electrode and rotating disk electrode, respectively, while PBS refers to phosphate buffered saline solution.

Following the evaluation of ORR activity in different media, it is also important to consider the role of ECSA. Although the values reported in Table 2 reflect the enhanced porosity and nitrogen doping of the N-CDCs, the ORR activity does not scale linearly with ECSA. This behavior suggests that the *C*_dl_, from which ECSA is estimated, may not exclusively reflect the density of electrochemically active sites. In our CDC materials, *C*_dl_ can be influenced by other contributions such as oxygen-containing surface groups or residual SiO_*x*_ species, both of which may induce pseudocapacitive or dielectric effects that increase the apparent *C*_dl_ without directly enhancing the ORR performance. Therefore, although the ECSA is a useful comparative descriptor of accessible surface area, it cannot be considered a sole or definitive predictor of ORR activity. In our case, the electrocatalytic performance is better rationalized by considering the synergy between the porous structure and surface chemistry, especially the incorporation of catalytically relevant nitrogen functionalities such as pyridinic-N and graphitic-N.

The stability of the electrocatalysts is studied by comparing the LSV curves before and after accelerated degradation tests performed in O_2_-saturated media (Figure S16). The stability decreases with increasing acidity of the electrolyte, with significantly better durability in alkaline media. This pH-dependent behavior is attributed to carbon framework oxidation and dopant loss, both of which are more severe in acidic environments due to higher corrosivity and active site protonation.^48^ In contrast, in alkaline conditions, the carbon oxidation is the primary degradation pathway, but the overall corrosion rate is considerably lower than in acidic media.^49,50^ The N-doped samples consistently outperform the Ref material across the different pH environments, reaching *E*_ONSET_^*^ (representing the potentials after degradation measurements) close to the initial values, confirming the beneficial role of nitrogen doping in enhancing ORR durability. Moreover, the 2D and 3D catalysts show similar catalytic stability, with *E*_ONSET_ decreasing by ≈20 and 70 mV, compared to 1D exhibiting a reduction of 50 and 110 mV for alkaline and neutral media, respectively (Table 2). This phenomenon is likely attributed to the lower nitrogen content and the excessive microporosity or insufficient meso- and macroporosity, resulting in an unbalanced pore structure (Table 1) of the 1D catalyst, accelerating its degradation across the varied media.^50,51^

Evaluating our findings with a Pt/C benchmark (Table 3), higher overpotentials are reached by the prepared CDC catalysts across the different media, indicating a more favorable ORR on Pt/C. However, the lower Tafel slopes exhibited by the N-CDCs in alkaline and neutral media indicate a more favorable kinetic process than benchmark Pt/C, as for the well-developed porous structure and nitrogen doping. Additionally, making a comparison to the results found in the literature (Table 3), similar conclusions are reported by Ratso et al.^52^ in alkaline media. In that study, N-doped CDCs were prepared from titanium carbides and carbonitrides, reaching surface areas comparable to those obtained in our study. Their findings concluded that despite the doping of CDCs with different nitrogen contents, the introduction of nitrogen heteroatoms has a minor effect on the ORR activity of CDCs in 0.1 M KOH, with the average pore size playing a significant role. This is in contrast to numerous studies in the catalysis field that demonstrate the enhancement of ORR activity of carbon-based materials through nitrogen doping, greatly introducing active sites that facilitate the O=O bond weakening.^2,11,53^ Likewise, several studies report this phenomenon, including the preparations of different carbon-based materials, such as activated carbons,^44,54^ carbon nanoplatelets,^9,55^ carbon nanospheres,^56^ and defective carbons,^24,57^ among others. However, CDCs are not considered, and only some studies related to N-doped CDCs with intermediate *S*_BET_ values (<500 m^2^ g^–1^) reveal the positive effect of nitrogen doping.^17,58^ Considering these findings, our results further support the conclusion of Ratso et al.,^52^ suggesting the existence of a threshold in the porous structure and the specific surface area beyond which the effect of nitrogen doping becomes negligible in CDC materials. Thus, the pore size and overall textural properties appear to be the key factors in determining the ORR activity of CDCs in 0.1 M KOH, in contrast to carbon-based materials prepared by other synthesis routes. Given the high concentration of defects found in the CDC materials, especially in the 2D sample, which is primarily associated with the processing route, no significant changes are observed in the catalytic activity performance. This phenomenon can be tentatively ascribed to the enhanced influence exerted by the elevated porous microstructure and the nitrogen doping of the prepared samples in comparison to the defect concentration.

To compare the electrocatalytic activity in the different pH media, Figure 5a presents the catalytic activity expressed as log­(*i*). This is represented as a function of the onset potential and is calculated from the Tafel slopes at a constant potential of 0 V vs RHE. As previously reported in other carbon-based materials doped with different heteroatoms, including transition metals,^64,65^ the activity increases with the pH media. In Figure 5b, the selectivity of the prepared materials in different pH media is studied through the number of electrons transferred. Here, our CDC materials exhibit an ORR governed by a two-step 2e^–^ pathway in the acidic medium, reaching *n* values of approximately 3 and moderate production of H_2_O_2_. This finding suggests that the O_2_ molecules are converted into H_2_O_2_, which is then converted into H_2_O. The ORR behavior is enhanced in a neutral medium as the oxygen reduction proceeds in the N-doped samples (1D and 3D) primarily via a direct 4e^–^ pathway. In this pathway, O_2_ is reduced into H_2_O, promoted by nitrogen incorporated into the prepared materials. In the final analysis, within the alkaline environment, the ORR mechanism reveals a multifaceted behavior, with the 2e^–^ pathway as the predominant feature. This observation is substantiated by the detection of high H_2_O_2_ levels (>63%, Table 2) in the N-CDC samples, which attain comparable peroxide production levels to those of the diverse prepared catalysts.

**Figure 5:**
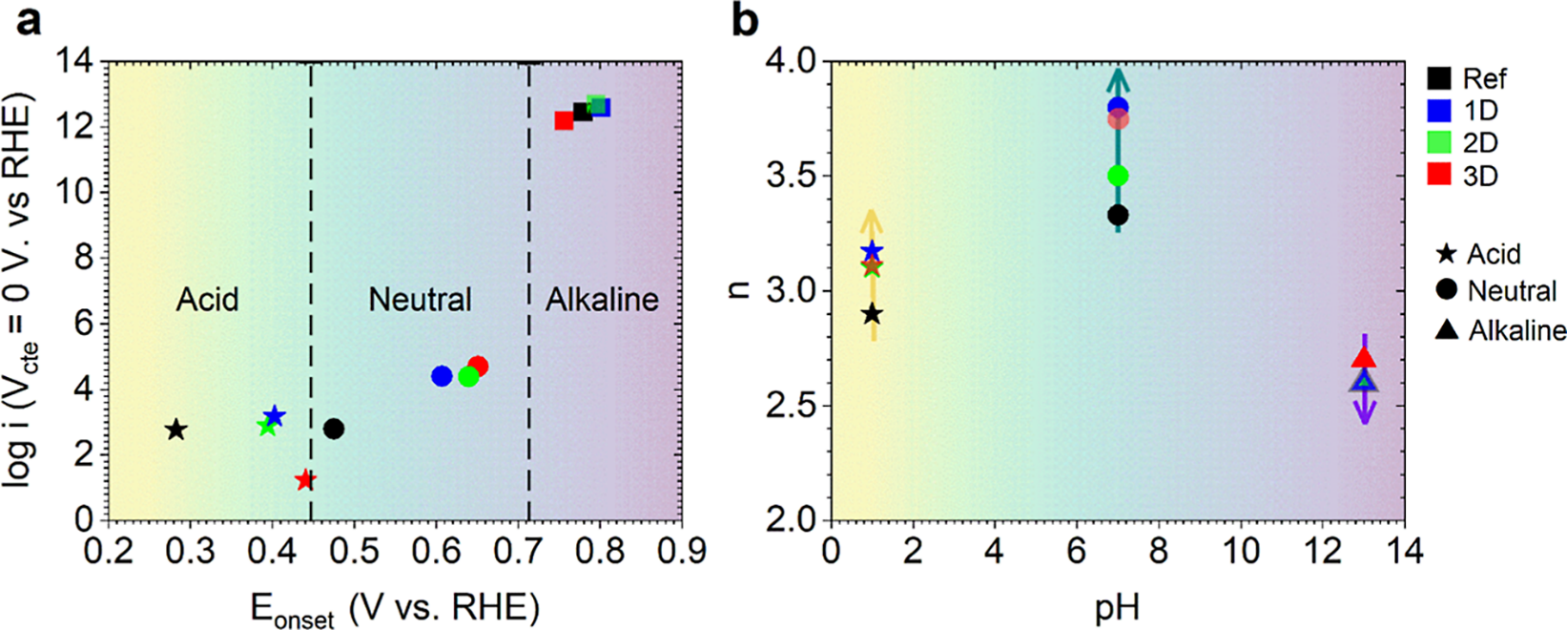
Analysis of the mechanism involved on the ORR performance of the CDCs materials across pH media. (a) Linear approximation based on the Butler–Volmer equation for the different media. (b) Comparison of the number of electrons transferred (*n*) with the pH media and samples.

Therefore, this research demonstrates the significant potential of the prepared CDC catalysts to promote the ORR in different media. Their performance is governed by the synergy between textural properties and effective nitrogen doping. To better understand the influence of the precursor chemistry on the final physicochemical and electrocatalytic properties of the CDC materials, a correlation between the structural features and the observed electrochemical performance is established. The AHPCS-derived undoped sample (Ref) exhibited a highly microporous structure with a large surface area (*S*_BET_ = 2057 m^2^ g^–1^), acting as a suitable porous framework for electrocatalysis. Additionally, the use of nitrogen-rich dendritic precursors allowed the incorporation of graphitic-N and pyridinic-N functionalities, both commonly associated with enhanced ORR activity, while also modulating the pore architecture. The hierarchically porous microstructures, which lead to elevated surface areas, primarily dictate the N-CDCs electrocatalytic activities in alkaline media, reducing the overpotentials for H_2_O_2_ generation and enabling better kinetics than the neutral and acidic conditions. In this medium, the different developed CDCs exhibit similar electrocatalytic behavior, largely attributed to their elevated *S*_BET_ values, with no apparent influence from nitrogen doping. Conversely, nitrogen doping plays a significant role in the acidic and neutral media, shifting the onset potentials toward positive values, indicating improved electrocatalytic activities. In neutral medium, 1D and 3D catalysts, both featuring superior *V*_micro_ and *S*_BET_ values, together with nitrogen doping fundamentally in the form of graphitic-N and pyridinic-N, show significant improvement in their electrocatalytic activities. These two nitrogen species are well-known to promote the ORR in the literature;^10,30,66^ however, further investigation of N-doped CDCs is imperative, particularly concerning the synthesis of CDCs with the presence of a single predominant N-species, as the effect of the different nitrogen functionalities in the field of carbon-based materials for the ORR is still a subject of ongoing debate.

Overall, these findings support the potential of the N-CDCs materials to serve as promising candidates for fuel cell applications operating in neutral media as well as in water treatment and hydrogen peroxide production in alkaline conditions.

## 4 Conclusion

We synthesize nitrogen-doped carbon-based catalysts through the development of novel dendritic structures that function as fundamentally nitrogen sources. The resulting materials show high specific surface areas (>2000 m^2^ g^–1^) and hierarchically porous structures, where micro-, meso-, and macropores coexist. The prepared catalysts are then evaluated in terms of their performance in ORR experiments in neutral, acid, and alkaline media. In the latter, the catalysts display the highest onset potentials (∼0.8 V vs RHE) and superior kinetics (∼58 mV dec^–1^). The textural properties of the prepared CDCs are crucial to their ORR performance under alkaline conditions, and nitrogen doping does not induce significant changes in this medium. In contrast, the N-doped materials show a significant improvement compared to the undoped material in both acid and neutral media. The 0.1 M PB solution is particularly promising, with the number of electrons transferred close to 4 and limited H_2_O_2_ production. The findings clearly show the significant potential of CDC materials in terms of their ORR performance. They can be synthesized with a tailored porous structure and controlled nitrogen doping, rendering them suitable for potential applications in the domains of fuel cells, water treatment, and electrocatalytic H_2_O_2_ production.

## Supplementary Material



## Notes

**Dendritic structures**: structural and thermal characterization; **Catalysts**: FE-SEM, STEM, N_2_ adsorption–desorption, and Raman spectra; **Electrochemical characterization**: CVs, ORR study in acidic media, performance comparison, and catalyst stability. **Tables:** survey XPS data, C 1s XPS data, and Raman data (PDF)
