# Supplementary material for: Synergistic Effect of Nitrogen Doping and Textural Design on Metal-Free Carbide-Derived Carbon Electrocatalysts for the ORR
Source: ACS Appl Mater Interfaces. 2025 Sep 9;17(38):53388–401. doi: 10.1021/acsami.5c10307 (PMC12464907; doi:10.1021/acsami.5c10307)
Supplement: Supplementary file 1 [file am5c10307_si_001.pdf]

SUPPORTING INFORMATION

Synergistic Effect of Nitrogen Doping and Textural Design on Metal-Free Carbide-Derived Carbon Electrocatalysts for ORR

Berta Pérez-Román [1,2]*, F. Javier Recio [3]*, Jesús López-Sánchez [1], Laura Pascual [4], M. Alejandra Mazo [1], Fernando Rubio-Marcos [1]*

[1] *Instituto de Cerámica y Vidrio (ICV-CSIC), C/Kelsen 5, 28049, Madrid, España*
[2] *Escuela de Doctorado UAM, C/Francisco Tomás y Valiente 2, 28049, Madrid, España*
[3] *Departamento de Química Física Aplicada, Facultad de Ciencias, Universidad Autónoma de Madrid, C/Francisco Tomás y Valiente, 7, Cantoblanco, 28049 Madrid, España*
[4] *Instituto de Catálisis y Petroleoquímica, CSIC, C/Marie Curie 2, 28049 Madrid, España*

**Authors to whom correspondence should be addressed. B.P-R, F.J R and F. R-M: Phone: +34 91 735 58 40 Fax: +34 91 735 58 43. E-mail: berta.perez@icv.csic.es, javier.recioc@uam.es* and *frmarcos@icv.csic.es*

Keywords: nitrogen doping, carbon-based materials, carbide-derived carbon, textural properties, hydrogen peroxide, oxygen reduction reaction

S1 Synthesis and characterization of nitrogen-containing dendritic structures

Three different dendritic structures (D1, D2 and D3) were designed with increasing branching and nitrogen content. The novel dendrons were prepared with varied bonding configurations to promote distinct nitrogen functionalities in the final CDC materials. To evaluate the effect of nitrogen incorporation, these branched polymeric structures were designed with highly reactive end-group functionalities (vinyl groups) to facilitate the polymerization with the AHPCS.

The D1 dendron was prepared following the synthesis procedure outlined in [1]. To increase the branching degree along the different dendritic structures, the synthesis strategy employed for the D2 and D3 dendrons, implies the necessity of a molecular structure with a halogen atom, to promote further nucleophilic aromatic substitution with commercial reactants. This molecular structure (di-1D) was obtained from the di-substitution of the cyanuric chloride involved in the preparation of D1, following the reaction pathway described as follows:

Materials

Cyanuric chloride, N, N-diisopropylethylamine (DIPEA), piperazine and Tris(2-aminoethyl)amine were provided from Sigma Aldrich (USA). The 4-vinylaniline (90%) was purchased from Fisher Scientific (USA). Thin layer chromatography (TLC) technique was employed to monitor the different reactions, using F_{254} aluminium oxide coated plates. Final products were obtained by purification process carried out by column chromatography, and employing silica gel 60 (60-120 mesh) obtained from Merck (USA).

Synthesis

i) Di-substituted molecule (di-1D): 6-chloro-N2,N4-bis(4-vinylphenyl)-1,3,5-triazine-2,4-diamine

Cyanuric chloride (16 g, 0.086 mol) and DIPEA (35 mL, 0.2 mol) were dissolved in (250 mL) of anhydrous tetrahydrofuran (THF), and stirred at RT under argon atmosphere. Then, (30.18 mL, 0.258 mol) of 4-vinylaniline was added dropwise, followed by heating the mixture under reflux for a period of 8 h. The obtained residue was purified by column chromatography using ethyl acetate/hexane (1:4) as eluent, obtaining a whitish solid product (> 85% yield).

ii) D2 dendron: 6,6'-(piperazine-1,4-diyl) bis(N2, N4-bis (4-vinylphenyl)-1,3,5-triazine-2,4-diamine

D2 was prepared by mixing (4 mL, 0.023 mol) of DIPEA and (688 mg, 0.008 mol) of piperazine in (250 mL) of anhydrous THF. Then, (5.5 g, 0.016 mol) of di-1D molecule was dissolved in anhydrous THF and incorporated to the previous mixture. The reaction was stirred, bubbled with argon and sealed in a Parr's vessel to ensure non-oxygen contamination. Then, the mixture was heated at 80 °C for 12 hours. The obtained product was obtained by performing a purification in column chromatography with dichloromethane/methanol (5:1) as eluent, obtaining a white product (> 85% yield).

iii) D3 dendron: N2-(2-(bis(2-((4,6-bis((4-vinylphenyl)amino)-1,3,5-triazine-2-yl)amino)ethyl)-N4,N6-bis(4-vinylphenyl)-1,3,5-triazine-2,4,6-triamine

In a flask, (6.95 mL, 0.04 mol) of DIPEA and (1.168 g, 0.008 mol) of Tris(2-aminoethyl) amine were dissolved in 250 mL of anhydrous THF. Then, (10 g, 0.028 mol) of di-1D molecule was dissolved in anhydrous THF and incorporated into the previous solution, obtaining a mixture that was stirred, bubbled in argon and sealed in a Parr's vessel to remove the oxygen content. The mixture was heated at 80 °C for 14 hours until the consumption of the di-1D molecule was detected by TLC. Once the chemical reaction was completed, the solvent was

removed by using a rotatory evaporator, and the final residue was obtained by washing the solid with ethyl acetate and acetone obtaining a yellowish solid (> 90% yield).

Characterization

The synthesized dendritic structures were characterized by means of nuclear magnetic resonance (NMR) analysis and mass spectrometry (MS) techniques to confirm the intended designed molecular architecture and to elucidate the characteristic bonds. The NMR study was conducted in a Bruker Avance Neo (USA) by diluting the samples in dimethyl sulfoxide (DMSO), and recording ^{1}H (500 MHz), ^{13}C (125 MHz) and ^{13}C NMR distortionless enhancement by polarization transfer 135° (DEPT-135) analyses. Mass spectrometry was conducted by diluting the samples in methanol (CH_3OH), in an Agilent (USA) 6520 model -Accurate-Mass LC/MS Q-TOF instrument. Thermal behaviour of the as-synthesized dendritic structures was also studied by means of thermogravimetry and differential thermal analysis (TG-DTA) (**Figure S7**). Measurements were conducted in a SDT Q600 TA instrument (USA) by heating the dendrons under an argon flow of 100 cm^3 min^{-1} up to 1000 °C, with a heating rate at 10 °C min^{-1}.

The NMR spectra of dendron D1 are displayed in our previous article [1], and information from D2 and D3 molecules is display as follows:

D2 dendritic molecule: ^{1}H NMR spectrum (**Figure S1**) displayed the following information: δ_H (ppm): H1 (3.9 (sbr, 8H)), H2 (5.16 (d, J_{cis}= 10.99 Hz, 4H), H3 ((5.74 (d, J_{trans}= 17.68 Hz, 4H)), H4 (6.70 (dd, J_{trans}= 17.60 Hz y J_{cis}= 10.93 Hz, 4H)), H5 (7.42 (d, J_{ortho}= 8.31 Hz, 8H)), H6 (7.78 (d, J_{ortho}= 8.15 Hz y 8H)), H7 (9.3 (sbr, 4H)). The ^{13}C-NMR spectrum (**Figure S2**) displayed the different carbon proton resonances (δ_C) and from the ^{13}C NMR DEPT-135 (**Figure S3**) technique, it is possible to elucidate between primary, secondary and tertiary carbons. Notice that resonances in the upright direction represent CH_3 or CH, and signals in the inverted orientation are related to CH_2. Therefore, the observed δ_C (ppm) corresponds to: C1 (43.19, CH_2), C2 (112.48, CH_2), C3 (120.30, CH), C4 (126.81, CH), C5 (131.28), C6 (136.86, CH), C7 (140.39), C8 (164.48), and C9 (165.12). From the MS, and through the measures of the mass-to-charge ratio of the ions, molecular mass was verified,, finding 357.18 m/z $[M^{+2}H]^{2+}$ and 713.356 $[M^{+}H]^{+}$, confirming that D2 dendron corresponds to $C_{42}H_{40}N_{12}$, with a molecular weight of 712.35 g mol^{-1}.

D3 dendritic molecule: ^{1}H NMR δ_H (ppm) (**Figure S4**): H1 (2.82 (d, J = 7.33, 6H)), H2 (3.51 (d, J = 8.70, 6H)), H3 (5.12 (d, J_{cis} = 10.93, 6H)), H4 (5.69 (d, J_{trans} = 17.63, 6H)), H5 (6.67 (dd, J_{trans} = 17.60 and J_{cis} = 10.93, 6H)), H6 (7.36 (d, J_{ortho} = 8.35, 12H)), H7 (7.80 (d, J_{ortho} = 8.26 y 12H)), H8 (9.09 (sbr, 3H)) and H9 (9.18 (sbr, 6H)). From the ^{13}C NMR spectrum (**Figure S5**) and ^{13}C NMR DEPT-135 (**Figure S6**), the following assignations were discerned; δ_C (ppm): C1 (39.25, CH_2), C2 (54.27, CH_2), C3 (112.21, CH_2), C4 (120.15, CH_2), C5 (126.67, CH), C6 (131.06), C7 (136.89, CH), C8 (140.67), C9 (164.56) and C10 (166.21). MS: calculated for $C_{63}H_{63}N_{19}$ was 1085 g mol^{-1}, finding 543.78 m/z $[M^{+2}H]^{2+}$ and 1086.56 $[M^{+}H]^{+}$ signals.

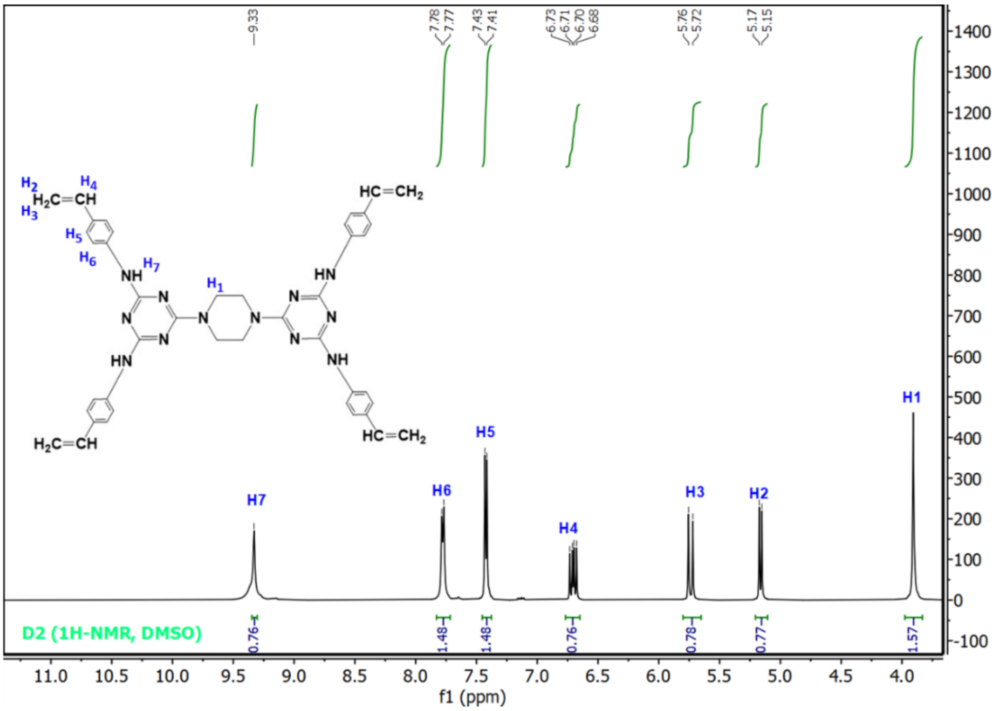

Figure S1| ^{1}**H-NMR spectrum of D2 dendritic molecule.** This spectrum reveals the distinctive proton signals (H1 to H7) corresponding to D2 dendron, elucidating the molecular structure of the novel dendritic molecule synthesized with the piperazine ring as a core centre.

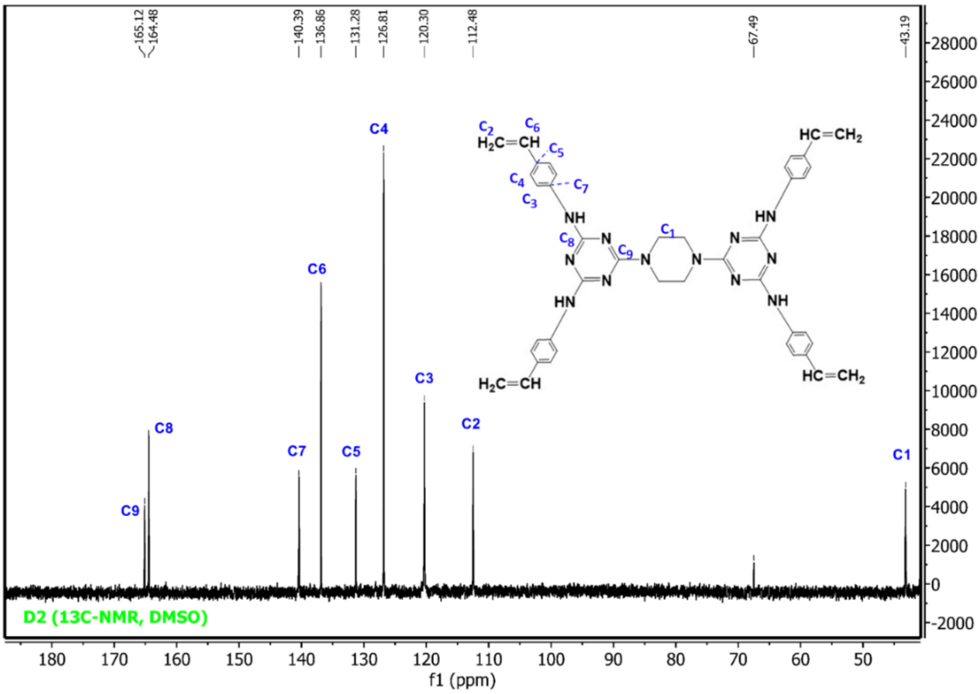

Figure S2| ^{13}**C-NMR spectrum of D2 dendron.** The spectrum shows the varied carbon resonances corresponding to the different carbon atoms of the dendritic molecule, labelled from C1 to C9.

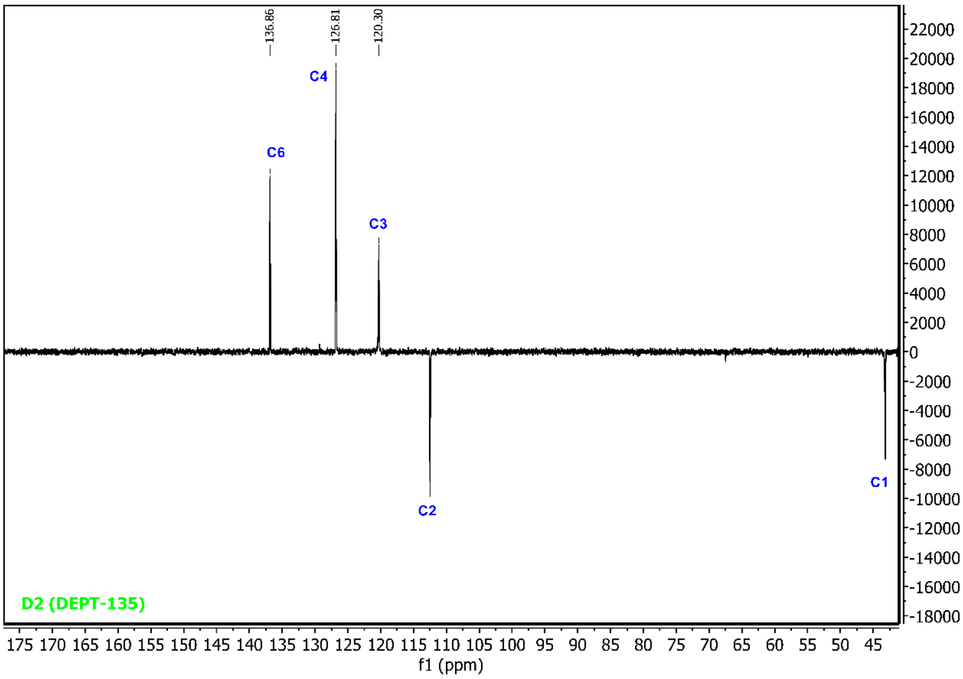

Figure S3 | DEPT-135 ^{13}C-NMR spectrum of D2 dendron. This spectrum offers detailed information about the carbon atoms, allowing differentiation between primary, secondary and tertiary carbons. Nobly, CH_2 resonances appear as downward signals, while CH and CH_3 appears in the upright direction. Quaternary carbons are not detected by this technique.

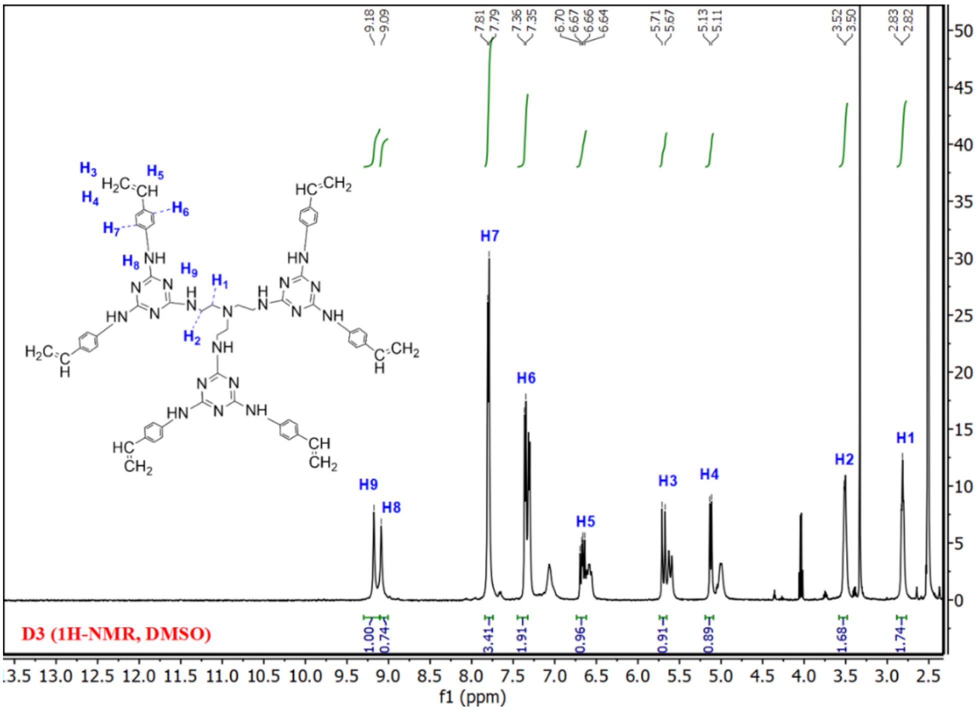

Figure S4 | ^{1}H-NMR spectrum of D3 dendron. This spectrum reveals the molecular intricacy of the proton resonances related to the D3 dendritic structure. Protons labelled as H3, H4 and H5 showed some signals at lower ppm values, related to the different magnetization experienced by these protons. These signals elucidate the different possible magnetic interaction between the different protons, highlighting the very complex D3 conformational structure. Notice the presence of several signals associated to the DMSO solvent used for the NMR measurement ((2.5 and 3.3 (H_2O) ppm), and other two signals (4.0 ppm and 7.2 ppm) both associated to ethyl acetate and toluene respectively, employed during the purification of the final product.

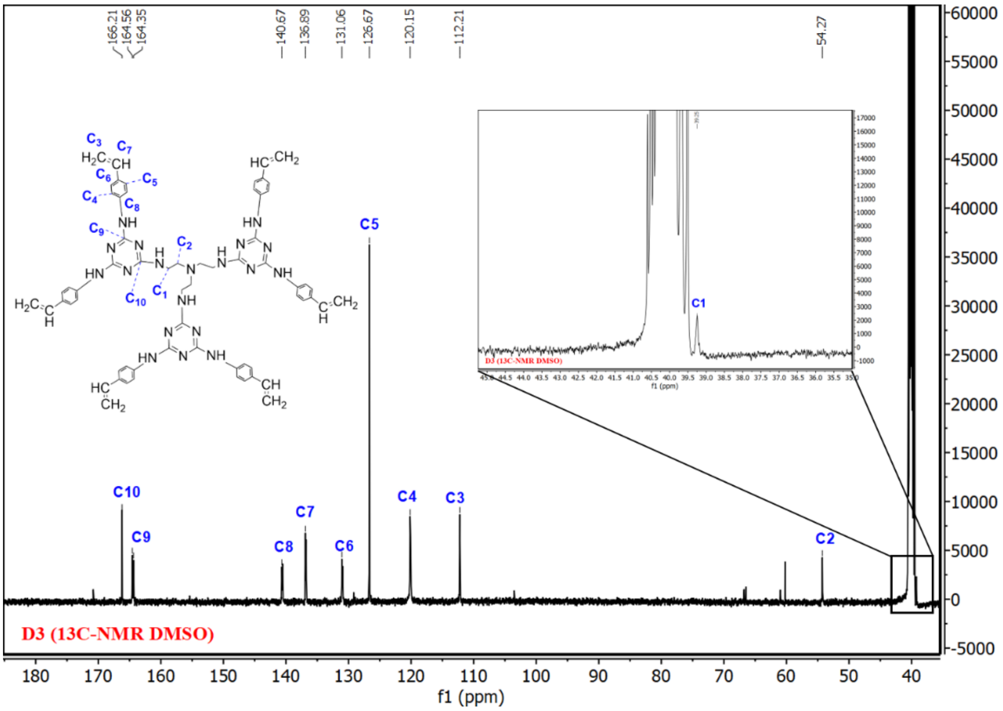

Figure S5| ^{13}C-NMR spectrum of D3 dendritic molecule. The spectrum reveals the carbon resonances corresponding to the D3 molecule (C1 to C10), coupled with some signals related to different solvents. The most intense signal observed at 39.5 ppm is related to the DMSO-d_6 employed as solvent for the NMR measurements, and the presence of other less intense signals due to solvents are also appreciated (ethyl acetate (170 and 60 ppm) and tetrahydrofuran (67 ppm)).

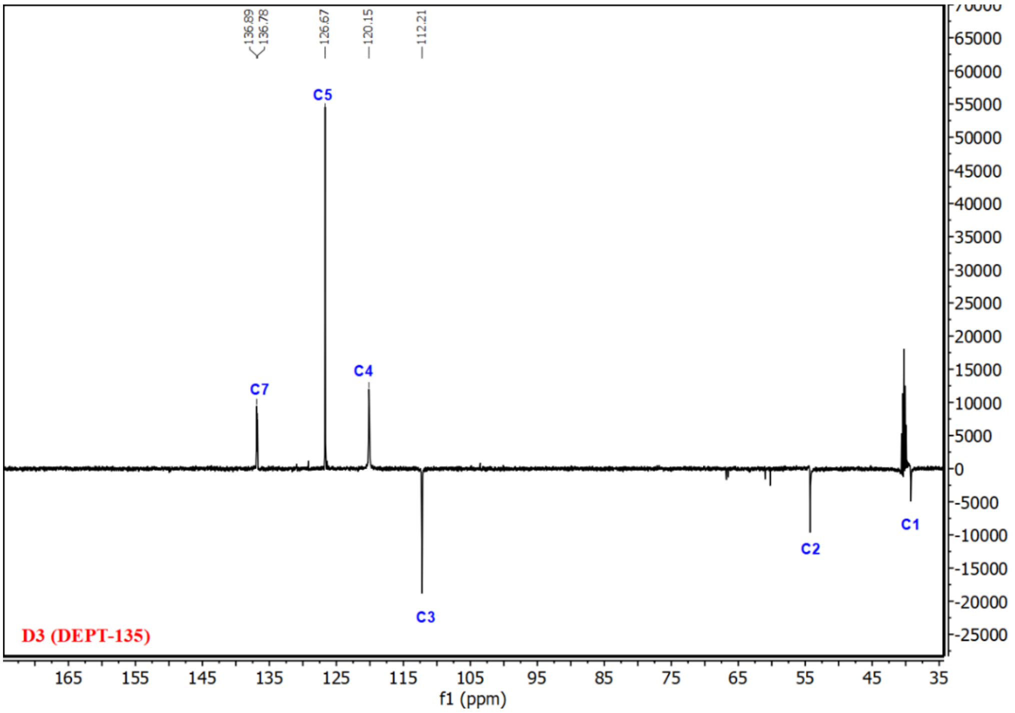

Figure S6| DEPT-135 ^{13}C-NMR spectrum of D3 dendritic structure. This spectrum displayed the presence of CH/CH_3 and CH_2 carbon in the D3 molecule, while the remaining carbon atoms in the structure are quaternary carbons. Notably, the upright signal at 39.5 ppm is related to the DMSO-d_6 used as solvent for the NMR measurements.

The TG-DTA spectra of dendrons are displayed in **Figure S7**, discerning different thermal behaviours. While D1 and D3 exhibit single thermal decompositions with major weight losses at ≈ 480 °C, the D2 dendron displays a dual thermal profile, with an additional weight loss at ≈ 261 °C. This earlier event is attributed to partial fragmentation of the dendron structure, releasing volatile fragments and creating internal "voids" during the initial crosslinking phase of the polymer-to-ceramic transformation. These voids evolve into larger pores after pyrolysis and chlorination, thus shifting the pore structure toward the meso/macroporous range and lowering S_{BET}.

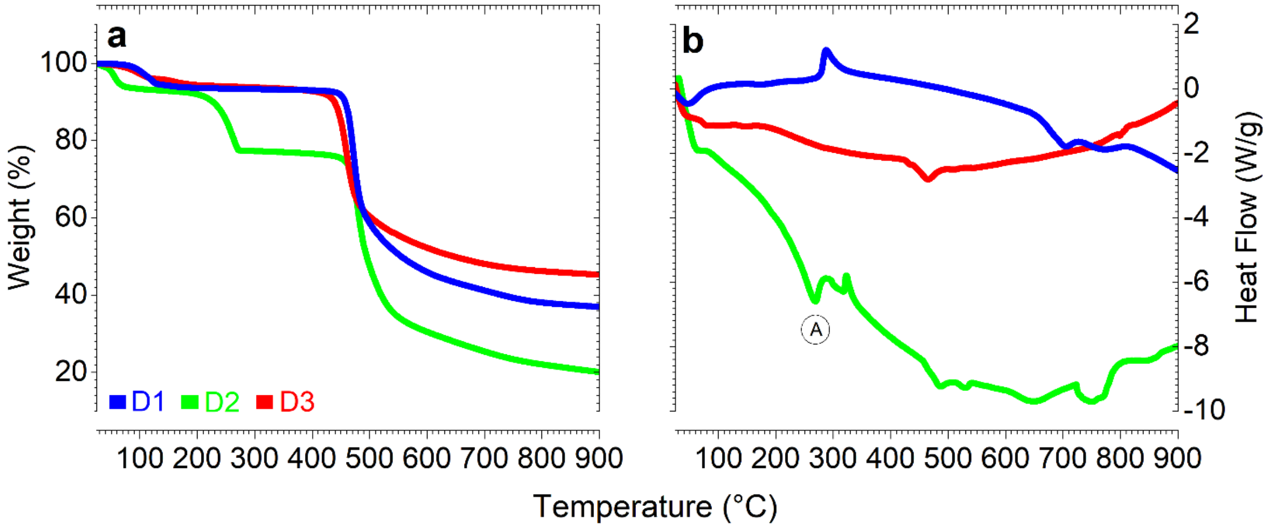

Figure S7| Thermal behaviour analysis of the dendritic structures. a. TG and **b.** DTA spectra of the different dendritic structures (D1, D2 and D3) from RT to 900 °C. Peak A denotes the endothermic peak attributed to the partial fragmentation of the D2 dendron.

S2 Microstructural and Structural Characterization of N-doped CDC materials

In this section, complementary information obtained from the characterization of the prepared materials is included. **Figure S8** displays the microstructural information obtained from FE-SEM and STEM microscopies, coupled with the elemental maps obtained from C and N elements. From the N_2 adsorption-desorption technique, the isotherm curves obtained for the prepared materials and their corresponding S_{BET} are included in **Figure S9**. Finally, carbonaceous materials are studied by means of Raman spectroscopy, displaying the obtained curves in **Figure S10**, as well as the ratio obtained between the characteristic bands D and G, namely as I_D/I_G.

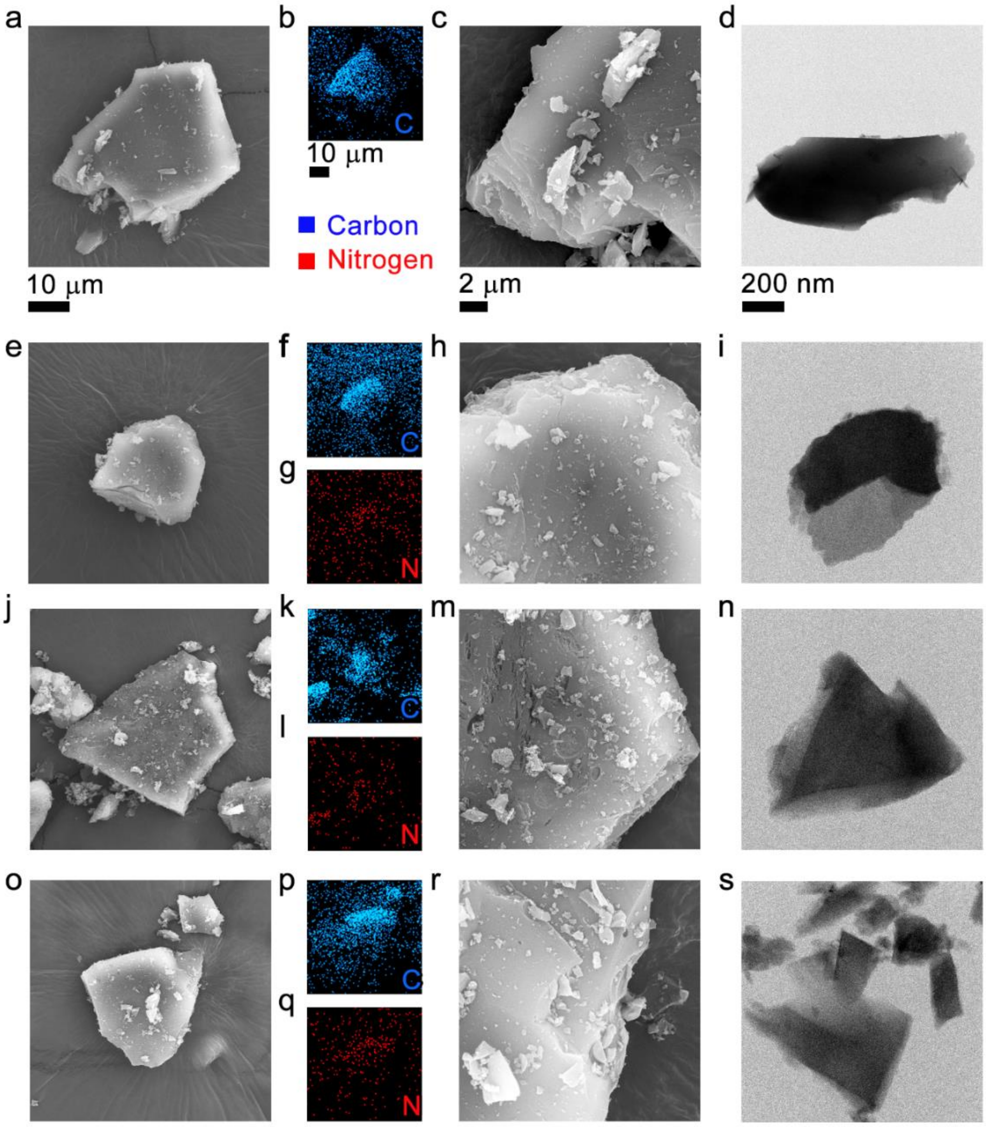

Figure S8| Microstructural characterization of the CDC materials by means of FE-SEM and STEM. (a) FE-SEM micrograph, (b) elemental mapping obtained by EDS, (c) high-magnification SEM micrograph and (d) TEM of Ref sample. (e), (j), (o) SEM micrographs, (f), (k), (p) carbon elemental maps (g), (l), (q) nitrogen elemental maps, (h), (m), (r) high-magnification SEM micrographs and (i), (n), (s) TEM images of the nitrogen-doped 1D, 2D and 3D CDC materials, respectively.

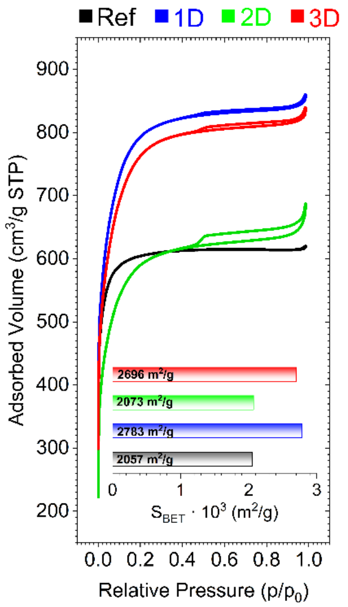

Figure S9| Textural analysis of the highly porous CDC structures. N_2 adsorption-desorption isotherms of the Ref sample, and the varied nitrogen-doped CDC materials (1D,2D and 3D), and their corresponding S_{BET}.

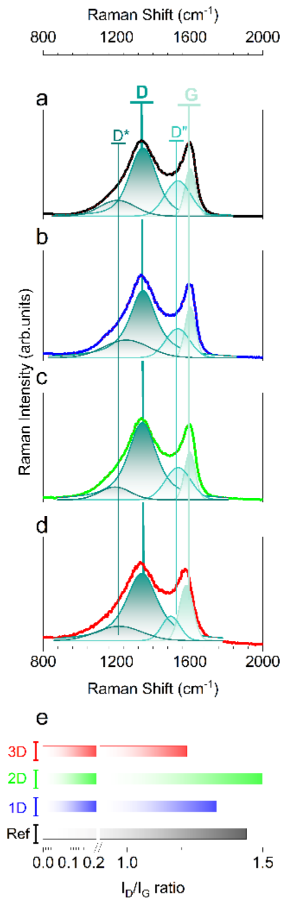

Figure S10| Raman study of the prepared CDC samples. Raman spectra obtained in the range of 800-2000 cm^{-1} for the (a) Ref (b) 1D (c) 2D and (d) 3D CDC samples. (e) I_D/I_G ratio obtained from the characteristic D and G bands of the prepared materials.

S3 Electrochemical characterization of N-doped carbon materials

In this section, information obtained from the electrochemical characterization of the synthesized materials is provided. CV performed in N_2-saturated electrolytes at scan rates of 5, 10, 20, 50 and 100 mV s^{-1} are displayed in **Figure S11-S13**, including measurements carried out in neutral, alkaline and acidic media, respectively.

The ORR performance of the prepared materials was also evaluated by LSV measurements, recorded in O_2-saturated electrolytes. **Figure S14** collects the behaviour of the prepared material in 0.1 M H_2SO_4 media, displaying the polarization curves obtained at 5 mV s^{-1}, the Tafel slopes, coupled with the H_2O_2 selectivity and the number of electrons transferred obtained from Koutechy–Levich equation. **Figure S15** includes a comparison between the variations of the onset (E_{ONSET}), the half-wave potential ($E_{1/2}$) and as well as the limiting current density (J_L) through the different media (neutral, alkaline and acid). The stability of the catalyst is also explored, displaying in **Figure S16** the LSV before and after accelerated degradation tests.

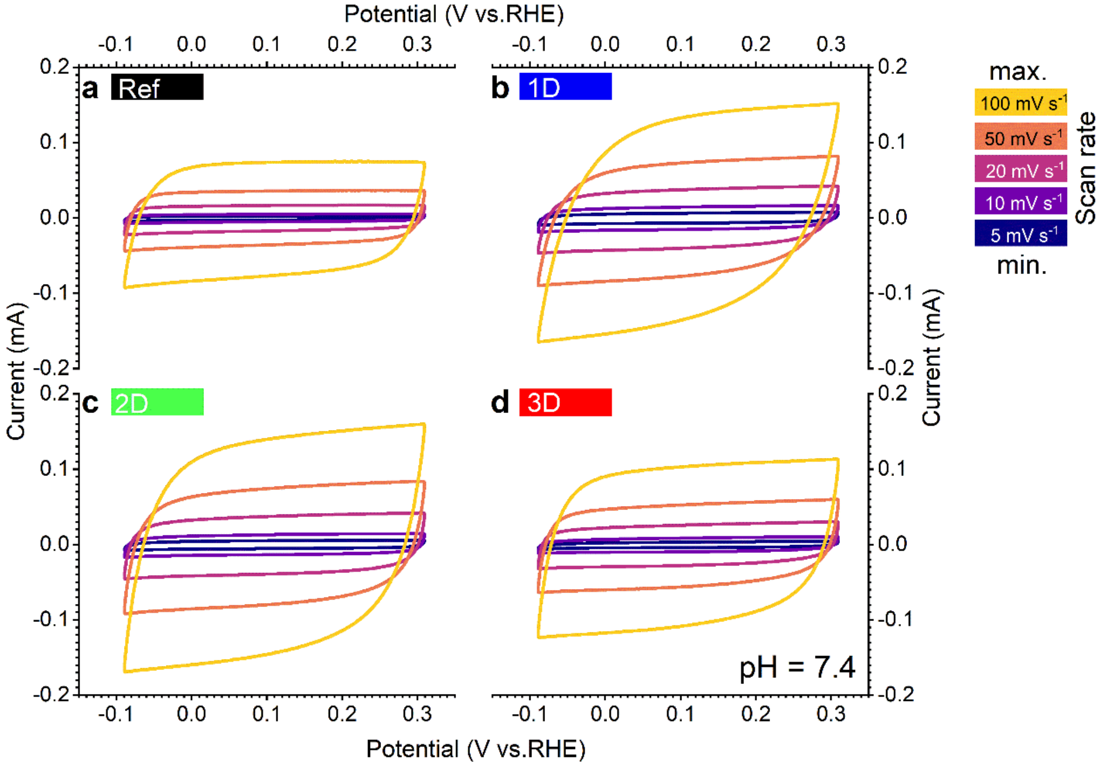

Figure S11| Electrochemical characterization *via* CV measurements obtained in N_2-saturated in 0.1 M PB solution. CV curves performed at scan rates of 5, 10, 20, 50 and 100 mV s^{-1} on the prepared CDC catalysts (a) Ref, (b) 1D, (c) 2D and (d) 3D CDC materials.

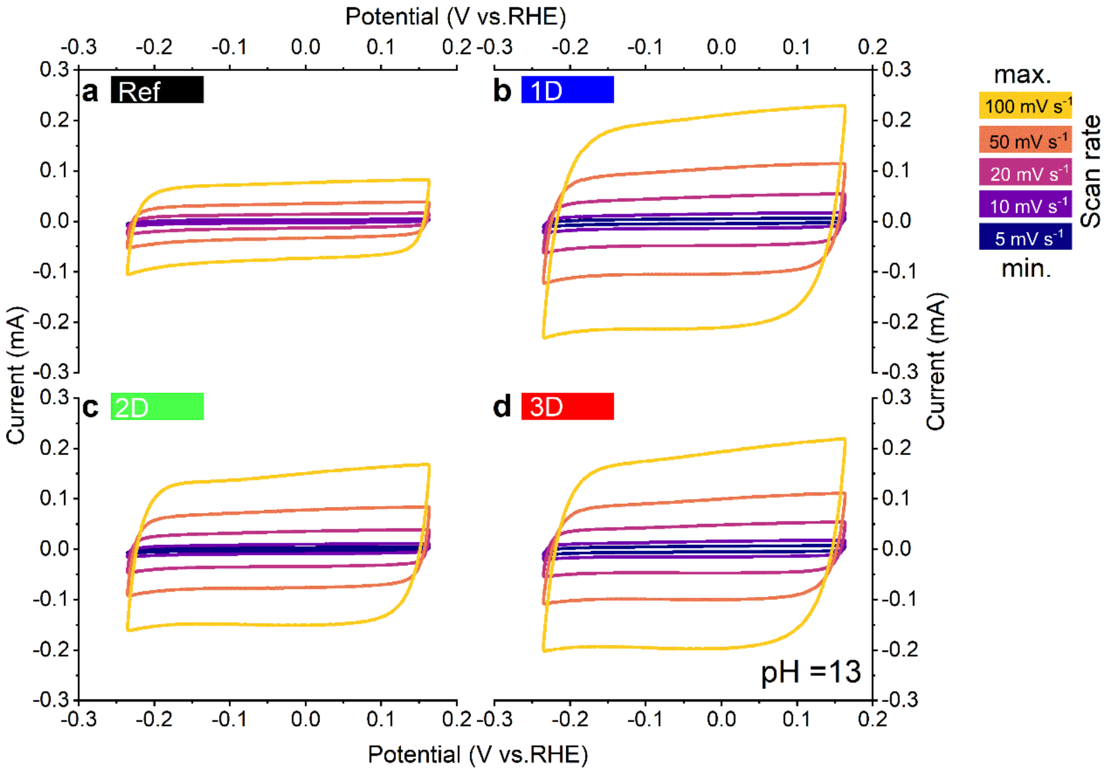

Figure S12| Electrochemical characterization *via* CV measurements obtained in N_2-saturated in 0.1 M KOH solution. CV curves performed at scan rates of 5, 10, 20, 50 and 100 mV s^{-1} on the prepared CDC catalysts (a) Ref, (b) 1D, (c) 2D and (d) 3D.

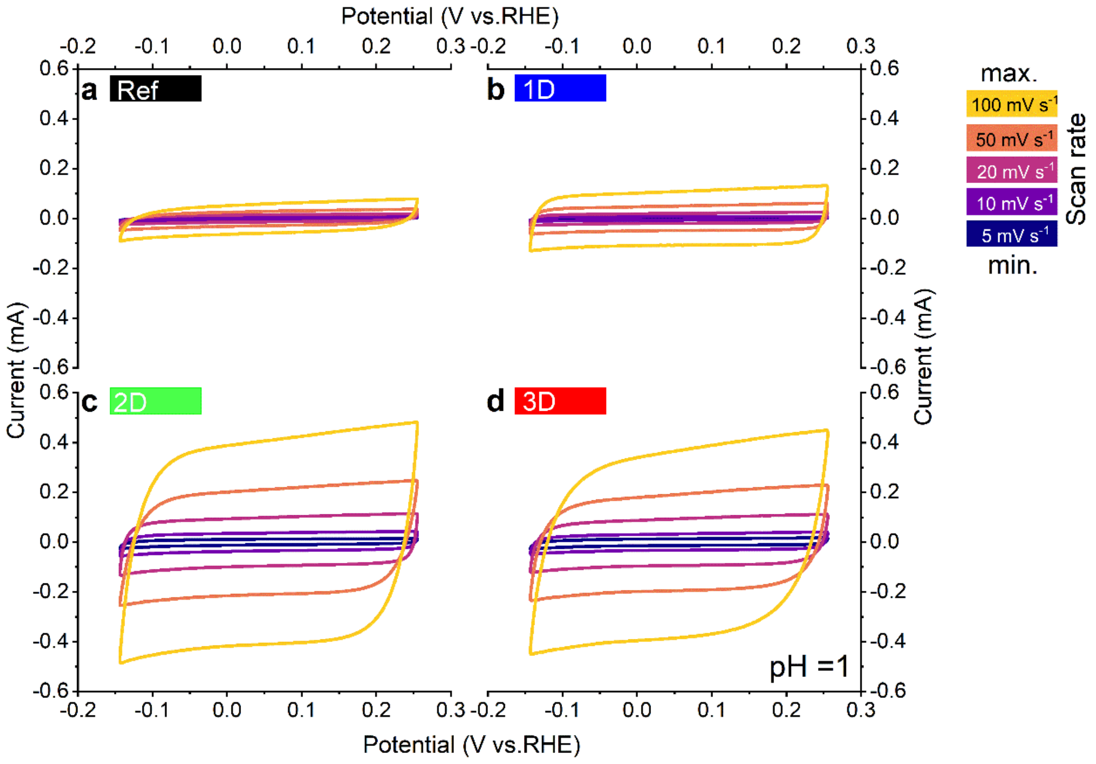

Figure S13| Electrochemical characterization *via* CV measurements obtained in N_2-saturated in 0.1 M H_2SO_4 solution. CV curves performed at scan rates of 5, 10, 20, 50 and 100 mV s^{-1} on the prepared CDC catalysts (a) Ref, (b) 1D, (c) 2D and (d) 3D.

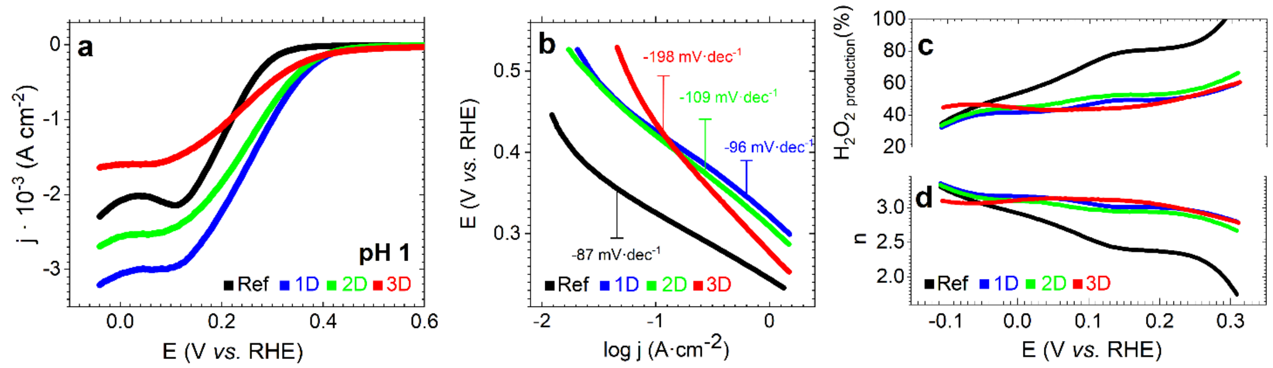

Figure S14| Electrocatalytic ORR performance in acid media. (a) LSV of the prepared catalyst in O_2-saturared 0.1 M H_2SO_4, (b) Tafel slopes, (c) H_2O_2 production and (d) number of electrons transferred. The LSV curves were recorded in a RRDE at a scan rate of 5 mV s^{-1}, and applying a rotation rate of 1600 rpm.

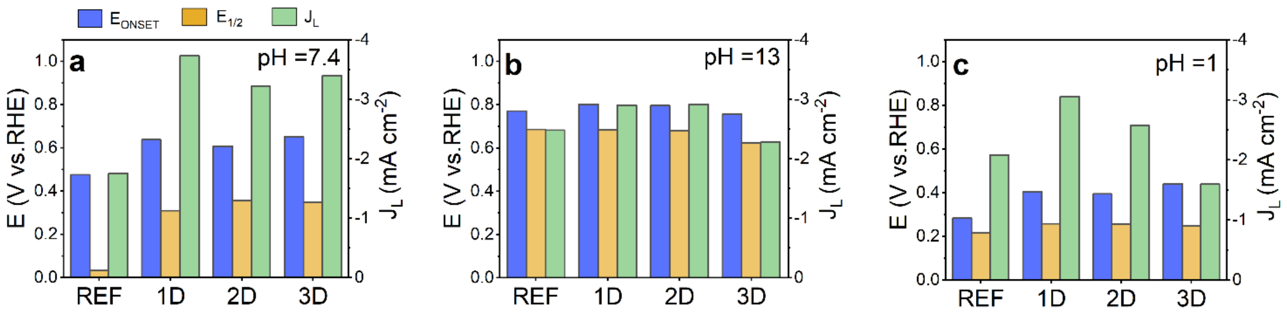

Figure S15| Effect of nitrogen doping into the electrochemical performance in different media. A comparison between the E_{ONSET}, $E_{1/2}$ and J_L is shown for the prepared CDCs materials in (a) 0.1 M PB, (b) 0.1 M KOH, and (c) 0.1 M H_2SO_4 media, respectively.

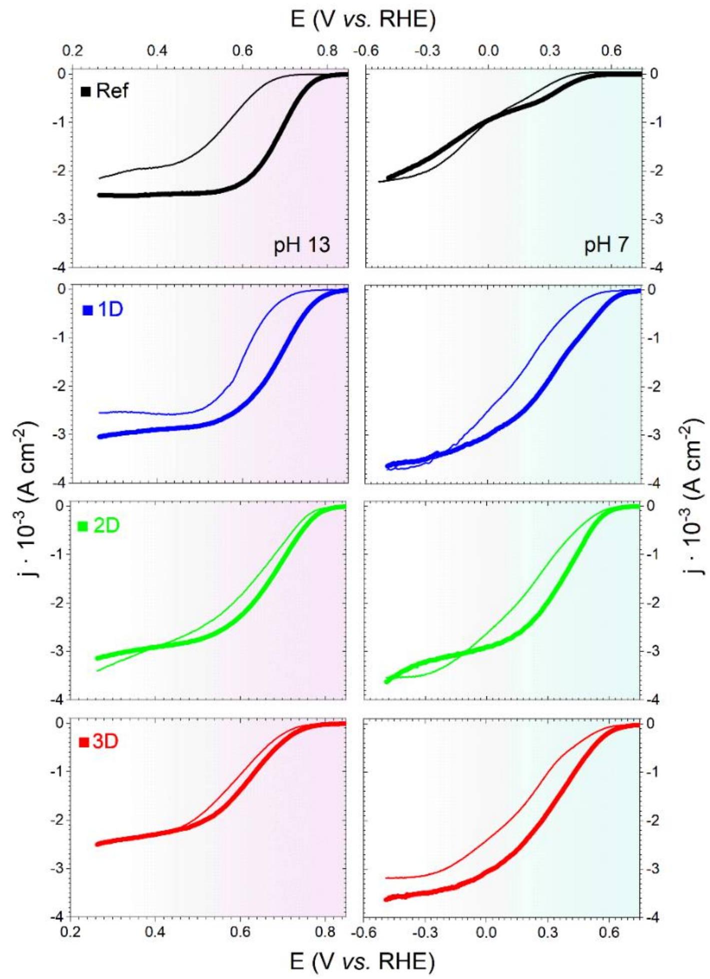

Figure S16 | Analysis of the catalyst stability. LSV curves of the catalysts before and after accelerated degradation test of 1000 CV cycles conducted in O_2-saturated electrolyte. Thicker and thinner lines represent LSV before and after degradation test, respectively.

S4 Tables

In this section, the information collected from the surface analysis conducted by XPS is displayed in **Table S1** with the elemental composition obtained from the survey, while **Table S2** elucidates the carbon speciation obtained from the high-resolution C1s XPS spectra. Additionally, Raman spectra were fitted by Voigt functions (**Table S3**), aiming to study the defect concentration through the assessment of I_D/I_G parameter.

Table S1. Surface elemental composition obtained from the XPS survey of the CDC samples.

Samples	Surface elemental composition from survey spectra wt %				
	C	N	O	Si	Cl
**Ref**	92.86	-	6.33	0.00	0.81
**1D**	87.53	5.24	6.41	0.00	0.82
**2D**	72.11	5.63	12.27	9.25	0.75
**3D**	88.00	5.46	4.78	0.91	0.85

Table S2. Data deconvolution obtained from the fitting of the high-resolution C1s spectra. Table includes position (Xc) in eV, Full-Width Half Maximum (FWHM), and area based-relative concentration (%) of the different carbon bonding. The ratio between Csp^3/Csp^2 is also included to elucidate the defect concentration.

	Ref				1D		
	Xc	FWHM	%		Xc	FWHM	%
Csp^2	284.6	1.1	45.0	Csp^2	284.5	1.3	38.9
Csp^3	285.3	2.7	36.4	Csp^3	285.3	2.5	42.9
C-N	-	-	-	C-N	287.6	2.3	6.6
C-O	289.3	4.9	18.6	C-O	290.1	3.9	11.6
Csp^3/Csp^2		0.81		Csp^3/Csp^2		1.10	
	2D				3D		
	Xc	FWHM	%		Xc	FWHM	%
Csp^2	284.5	1.2	33.3	Csp^2	284.6	1.3	35.0
Csp^3	285.2	2.4	44.9	Csp^3	285.1	2.6	40.0
C-N	287.5	2.2	5.8	C-N	287.3	3.5	14.8
C-O	289.8	4.6	16.0	C-O	290.6	3.5	10.3
Csp^3/Csp^2		1.35		Csp^3/Csp^2		1.14	

Table S3. Structural information obtained from the Voigt deconvolution performed in the Raman spectra. Table collects the information of each fitted spectrum, including the position of each band (Xc), Area, Intensity, and I_D/I_G parameter.

		Deconvolution of the Raman Modes by Nonlinear Curve Fit (Voigt)				
		Band D*	Band D	Band D''	Band G	I_D/I_G
Ref	Xc (cm^{-1})	1206.53	1345.12	1536.95	1603.69	1.44
	Area	699.32	2808,04	1136.72	647.02	
	Intensity	2.87	12.43	6.42	8.61	
1D	Xc (cm^{-1})	1253.26	1345.55	1535.04	1604.18	1.33
	Area	1001,23	3301,30	873,11	681,75	
	Intensity	3.23	12.28	5.30	9.23	
2D	Xc (cm^{-1})	1196.34	1340.74	1523.96	1600.12	1.51
	Area	378,96	4111,61	851,11	753,98	
	Intensity	1.99	14.26	4.79	9.45	
3D	Xc (cm^{-1})	1214.55	1339.31	1497.79	1582.12	1.22
	Area	813,19	3691,36	552,69	952,31	
	Intensity	2.60	12.27	4.42	10.07	

Supplementary references

(1) Pérez-Román, B.; Merchán del Real, A.; Rubio, J.; Mazo, M. A.; Rubio-Marcos, F. Innovative Strategies for Nitrogen-Incorporating Silicon Oxycarbide-Based Preceramic Polymer Synthesis. *Mater. Adv.* **2024**, *5* (5), 2040–2056. https://doi.org/10.1039/d3ma00898c.
